# Critical evaluation of short, long, and hybrid assembly for contextual analysis of antibiotic resistance genes in complex environmental metagenomes

**DOI:** 10.1038/s41598-021-83081-8

**Published:** 2021-02-12

**Authors:** Connor L. Brown, Ishi M. Keenum, Dongjuan Dai, Liqing Zhang, Peter J. Vikesland, Amy Pruden

**Affiliations:** 1 Genetics, Bioinformatics, and Computational Biology, Virginia Tech, Blacksburg, VA 24060 USA; 2 Department of Civil & Environmental Engineering, Virginia Tech, Blacksburg, VA 24060 USA

**Keywords:** Computational biology and bioinformatics, Microbiology, Environmental sciences, Engineering

## Abstract

In the fight to limit the global spread of antibiotic resistance, the assembly of environmental metagenomes has the potential to provide rich contextual information (e.g., taxonomic hosts, carriage on mobile genetic elements) about antibiotic resistance genes (ARG) in the environment. However, computational challenges associated with assembly can impact the accuracy of downstream analyses. This work critically evaluates the impact of assembly leveraging short reads, nanopore MinION long-reads, and a combination of the two (hybrid) on ARG contextualization for ten environmental metagenomes using seven prominent assemblers (IDBA-UD, MEGAHIT, Canu, Flye, Opera-MS, metaSpades and HybridSpades). While short-read and hybrid assemblies produced similar patterns of ARG contextualization, raw or assembled long nanopore reads produced distinct patterns. Based on an in-silico spike-in experiment using real and simulated reads, we show that low to intermediate coverage species are more likely to be incorporated into chimeric contigs across all assemblers and sequencing technologies, while more abundant species produce assemblies with a greater frequency of inversions and insertion/deletions (indels). In sum, our analyses support hybrid assembly as a valuable technique for boosting the reliability and accuracy of assembly-based analyses of ARGs and neighboring genes at environmentally-relevant coverages, provided that sufficient short-read sequencing depth is achieved.

## Introduction

Antibiotic resistance is one of the greatest health threats of the twenty-first century. Within the United States, it is conservatively estimated that 2.8 million people are sickened each year by antibiotic resistant infections due to antibiotic resistant pathogens and 35,000 die as a result^1^. Understanding how antibiotic resistance amplifies and disseminates is a key challenge so that effective mitigation can take place. The environment has been identified as a potential source of antibiotic resistance dissemination relevant to human health though further characterization is needed^2^. The need to better quantify environmental sources and pathways; including sewage, wastewater effluents, biosolids, animal manure, and surface runoff, of antibiotic resistance is gaining increasing attention in the global battle against the spread of antibiotic resistance spread. Next generation sequencing (NGS) is emerging as a powerful tool in this battle, making it possible to comprehensively characterize environmental metagenomes, including the full range of antibiotic resistance genes (ARGs) and mobile genetic elements (MGEs). A typical workflow for characterizing environmental antibiotic “resistomes” (i.e., total ARGs and MGEs) involves collecting the sample of interest, extracting DNA, library preparation, and sequencing on an Illumina platform^3–5^. This approach produces millions of short reads (typically 100–150 bp long) of DNA derived from the sample of interest that can be directly compared against publicly available databases to identify ARGs and other genes of interest and their relative abundances.

As more environmental metagenomes continue to be evaluated, the sheer diversity of ARGs that exist in various environments has been eye-opening^6–8^. However, the length of short reads precludes the ability to answer key research questions that must be addressed to better inform monitoring and mitigation of antibiotic resistance in the environment. In particular, the contextualization of ARGs, i.e., predicted carriage on MGEs and identification of host bacteria, especially pathogens, is essential information needed to characterize the drivers of the dissemination and attenuation of antibiotic resistance. The assembly of short reads to form longer stretches of DNA or “contigs” is one way to achieve this goal. This can be achieved via several available short-read assembly algorithms. Alternatively, new technologies such as single-molecule real time sequencing, performed via the Oxford Nanopore or Pacific Biosciences sequencing platforms, enable generation of extremely long-reads. Often DNA sequences exceeding 20 kilobases (kb) in length can be produced^9^, making it possible to directly capture associations between ARGs with hosts or MGEs without the need for assembly^10^. However, long-read sequencing, at present, is more costly and has higher error rates^11^.

Reference-independent (de novo) assembly of metagenomes has become standard practice for contextualizing environmental-borne antibiotic resistance because these environments are typically poorly represented in publicly-available databases and thus reference genomes are not available^12–16^. While historically de novo assembly methods relied on overlap layout consensus (OLC)^17^, such an approach is intractable for the millions of reads generated by NGS platforms^17^. In contrast, most modern short-read assemblers rely on de Bruijn graphs (dBg)^18^, which are *n-*dimensional directed graphs that represent overlaps of length *k-*mers extracted from short reads as nodes and connections between adjacent *k-*mers as vertices. However, assembly using dBgs poses additional computational challenges, which have been recently reviewed by Ayling et al.^19^. Briefly, these challenges include the selection of an appropriate *k-*mer size, the handling of uneven genome abundance, resolution of ambiguity in the dBg introduced from related strains or sequencing errors, and computational challenges associated with handling millions of reads.

While long-reads capture longer stretches of DNA, it remains desirable to assemble the reads into metagenome-assembled genomes (MAGs), enabling in-depth profiling of microbial taxa within a sample^20–22^. Compared to short-read assembly pipelines, there are relatively few options available for assembly of long-read-derived metagenomes. While there are no tools specifically dedicated to long-read metagenome assembly, Canu^23^, metaFlye^24^, and Miniasm^25^ have been applied in prior investigations^22,26,27^. Such approaches rely on overlap layout consensus methods that join and merge overlapping reads into a contig that reflects the consensus of the multiple reads^26,28,29^.

More recently, hybrid assembly strategies have emerged^30,31^, leveraging the value of both short and long-reads. Hybrid assembly has the potential to enhance detection and contextualization of ARGs in environmental metagenomes by combining the high accuracy and greater depth provided by short reads with the increased length of long-reads, which may span repeat-rich regions that are difficult to assemble for dBg-based approaches. For instance, OPERA-MS; which relies on MEGAHIT^32^ to assemble short-reads, an OLC method to extend over gaps between short reads using corresponding long-reads, and a reference genome-based binning step, has been used to resolve strain-level associations between ARGs and MGEs^30^. However, such hybrid assembly approaches have not been critically evaluated in the context of complex environmental samples. Environmental samples are especially challenging for metagenome assembly due to the presence of tens of thousands of microbial species (e.g., Johnston et al.^33^), including many closely-related strains^34^ and low abundance species with similar depth profiles that are difficult to distinguish^35,36^.

The objective of this study was to assess and compare the performance of short, long, and hybrid read assembly pipelines for the purpose of contextualizing ARGs in complex environmental samples^37^. Using a combination of real data from ten globally-sourced wastewater metagenomes sequenced both with Nanopore MinION and Illumina technologies and simulated reads, we evaluated assembler-driven differences in ARG contextualization and compared the results of assembly leveraging one or both sequencing technologies. We further quantified assembly error in recovering and accurately assembling an in silico spiked genome from within the samples. The results outline critical considerations for the application of shotgun metagenomics and assembly towards advancing understanding of key ecological processes driving environmental dissemination and attenuation of antibiotic resistance.

## Methods

### Sample collection and processing

Sampling was conducted at two sites (influent (Inf) and activated sludge (AS)) at 5 different WWTPs from five countries (India (IND), Hong Kong (HKG), United States of America (USA), Switzerland (CHE), and Sweden (SWE)), between March 2016 and January 2017 as reported in Li et al.^38^. WWTP capacities ranged from 2.6 to 66 million gallons per day, and all plants relied on conventional AS treatment. Sample collection and processing was conducted using standardized protocols that were validated for preservation and stability of samples during international shipment^39^. Briefly, influent and AS samples for molecular analysis were collected at each WWTP in sterile polypropylene containers. All samples were transported to the laboratory on ice. Samples were processed within 12 h of collection. Illumina samples were processed in triplicate and aliquots of each sample were concentrated onto 0.22 µm pore size mixed cellulose ester membranes (Millipore, Billerica, MA) until clogging. Filters were preserved in 50% ethanol and shipped to Virginia Tech on ice packs. Upon arrival, filters were frozen at − 20 °C until DNA extraction. To prepare for DNA extraction, filters were aseptically torn into 1 cm^2^ pieces using sterile forceps and transferred to DNA extraction tubes. DNA was extracted using the FastDNA SPIN Kit for Soil (MP Biomedicals, Solon, Ohio) according to the manufacturer's instructions. The resulting DNA was purified with a genomic DNA clean kit (Zymo Research, Irvine CA), and quantified with a Qubit Fluorometer (ThermoFisher Scientific, Waltham, MA).

### Short reads

Composite DNA samples were prepared by pooling triplicates by equal DNA mass. Influent and IND-AS samples were composited and prepared for sequencing using TrueSeq library preparation (Illumina, San Diego, California). These samples were then sequenced through Illumina HiSeq 2500 using 2 × 100 paired-end reads. Sequencing was performed at the Virginia Tech Biocomplexity Institute Genomic Sequencing Center (Blacksburg, VA). The remaining AS activated sludge samples were composited and prepared for sequencing using the NEB Ultra II DNA Library Prep kit for Illumina library preparation (New England Biolabs, Ipswich, Massachusetts). These samples were then sequenced on an Illumina NextSeq500 using 2 × 75 paired-end reads. Sequencing was performed at the Scripps Research Institute Next Generation Sequencing Core Facility Microarray Core Facility (La Jolla, California). The resulting summary statistics, sample information and base pairs sequenced are displayed (Table 1).

**Table 1:** Unassembled read data and treatment plant capacity.

Location	Treatment process	SampleID	Treatment plant capacity (MGD)	Illumina platform data (length, platform, sequencing facility)	# of Illumina reads	Total bp sequenced (Illumina)	Average length of raw nanopore reads	N50	# of raw nanopore reads	Total base pairs sequenced (Nanopore)
India	Influent	IND-In	14	HiSeq 2500, 2 × 100, VT BI	12,961,380	2.59E + 09	1496	1973	8.33E + 05	1.25E + 09
Hong Kong	Influent	HKG-In	66	HiSeq 2500, 2 × 100, VT BI	10,069,772	2.01E + 09	2278	3868	2.34E + 06	5.34E + 09
Sweden	Influent	SWE-In	26	HiSeq 2500, 2 × 100, VT BI	11,973,421	2.39E + 09	4399	6292	5.47E + 05	2.41E + 09
Switzerland	Influent	CHE-In	32.6	HiSeq 2500, 2 × 100, VT BI	15,577,543	3.12E + 09	4141	5950	7.59E + 05	3.14E + 09
USA	Influent	USA-In	6	HiSeq 2500, 2 × 100, VT BI	13,534,261	2.71E + 09	1918	3211	7.19E + 05	1.38E + 09
India	Activated sludge	IND-AS	14	HiSeq 2500, 2 × 100, VT BI	13,520,740	2.7E + 09	2834	4600	1.50E + 06	4.26E + 09
Hong Kong	Activated sludge	HKG-AS	66	NextSeq500, 2 × 75, Scripps	8,124,534	1.22E + 09	2022	3389	3.00E + 06	6.07E + 09
Sweden	Activated sludge	SWE-AS	26	NextSeq500, 2 × 75, Scripps	11,953,275	1.79E + 09	2364	3828	1.07E + 06	2.53E + 09
Switzerland	Activated sludge	CHE-AS	32.6	NextSeq500, 2 × 75, Scripps	14,454,216	2.17E + 09	2896	4707	5.50E + 05	1.59E + 09
USA	Activated sludge	USA-AS	6	NextSeq500, 2 × 75, Scripps	8,541,529	1.28E + 09	3714	5973	5.47E + 05	2.03E + 09

### Long reads

Long-read metagenome samples were sequenced to obtain equivalent total basepairs relative to what was captured in the corresponding short-read sequencing run*.* Samples were pooled with equal mass (500 ng) from triplicate samples, and characterized with a NanoPhotometer (Implen, Westlake Village, CA) to examine purity (target OD 260/230 = 2.0–2.2, OD 260/280 > 1.8). If required, further concentration (target > 22 ng/μl), or purification of pooled DNA, was conducted using the genomic DNA clean kit. No degradation during the purification step was confirmed when checking DNA size distribution using DNA Screen Tape (Agilent, Santa Clara, CA). Nanopore sequencing was conducted using at least 1000 ng of DNA for each library preparation. Each sample was barcoded and used to prepare its own individual library (no multiplexing), and sequenced with a new flow cell (R9.0 or R9.4) in a MinION sequencer. Sequences were collected without real time base calling. Table 1 contains the resulting summary statistics, sample information and base pairs sequenced.

### Selected metagenomic assemblers and quality evaluation

Sevem assemblers were selected (MEGAHIT, IDBA-UD, metaSPAdes, Canu, metaFlye, HybridSpades, OPERA-MS) based on the popularity of use and their potential to assemble highly complex environmental samples^40,41^. Short reads were assembled using the recommended settings for IDBA-UD (September 2019 release)^42^, metaSPAdes (v.3.14.1) and MEGAHIT (v1.2.8)^32^. Though not explored in the present work, parameter settings can influence results and should be adjusted depending on the specific research objectives and questions. Nanopore reads were assembled using Canu (v 1.8)^23^ and metaFlye (v2.6)^24^. MetaFlye was run using predicted genome sizes of 10 mb, 1 gb, and 10 gb, but was found to produce similar results (not shown), a genome size of 1 gb was chosen. Canu was run using the recommended settings for metagenomic samples provided in the supporting documents for v1.8^43^. Hybrid assemblies were created using HybridSPADES (v.3.14.1)^31^ and OPERA-MS (v 0.8.3)^30^. Commands used for conducting assemblies may be found in (Supplementary Information 2). All evaluations were performed on contigs provided by the various assemblers as well as unassembled Illumina and Nanopore reads. Assemblies produced in this study can be found in BioProject PRJNA527877 in NCBI GenBank (Sample Data and SRAs can be found in Supplementary Table S1, https://www.ncbi.nlm.nih.gov/bioproject/PRJNA527877).

### Gene annotation

Nanopore reads and assemblies were analyzed for co-occurring ARGs, MGEs, and pathogen gene markers using MetaCompare^44^, which utilizes an ORF predictor, Prodigal^45^, followed by Diamond^46^ alignment against CARD^47^, ACLAME^48^, and PATRIC^49^. Contextualization results were visualized using a Bray Curtis dissimilarity matrix to generate non-metric multidimensional scaling (NMDS) plots.

### In silico read spiking

To assess the accuracy of assemblies, simulated short reads and long -reads were generated from the *Marinobacter hydrocarbonoclasticus* ATCC 49840 genome (NCBI-ID: NC_017067.1). This organism was chosen because it is a saltwater Gammaproteobacterium absent from the sequenced wastewater samples, as validated by no hits using Nucmer^50^, to align assemblies from non-spiked samples against the reference genome (Supplementary Figure S1). Short reads were generated using ART^51^ and Nanopore reads were generated using NanoSim (v 2.5.1)^52^. Briefly, short reads from HiSeq2500 (100 bp) or NextSeq (75 bp) were simulated and spiked into USA-AS and USA-Inf samples respectively at 1 × , 5 × , 10 × , and 50 × coverage of the reference genome in silico (Table S2). Nanopore reads of the *Marinobacter hydrocarbonoclasticus* genome were generated using a custom error profile trained from a 100,000 read subset of a sequencing run of the Zymo mock microbial community on an R9.4 flowcell (ENA-ID: ERR3152364)^53^. Simulated reads were then generated using the distribution of Nanopore read sizes from the USA-Inf and USA-AS at 0.1×, 1×, 3× (Inf), or 5 × (AS) coverage (Supplementary Table S3). Reads were differentially spiked at 3 times or 5 times coverage so that simulated reads would constitute no more than 20 percent of the total number of reads (Supplementary Table S4).

### Detecting presumptive misassemblies

To detect presumptive misassemblies, we aligned contigs to the reference genome using Nucmer (v3.3)^50^ and classified contigs as correct or incorrect assemblies using a custom algorithm in R (Supplementary Fig. S1). Briefly, we removed background contigs from spiked samples (background defined as identity < 95% and alignment length < 210 bp). These criteria were selected because they removed > 97% of the non-spiked sample assemblies that aligned with the reference genome using Nucmer (Supplementary Fig. S2). We then separated contigs into presumptive correct and incorrect assemblies for verification. Initial presumptive misassemblies were those with alignments that extend to less than 99% of the contig. Presumptive correct assemblies were those with alignments that extend to at least 99% of the length of the contig but less than or equal to 100%. Contigs that did not meet these criteria were then checked to determine if the sum of query coverages of its aligned regions from multiple aligned regions was greater than or equal to 99%, but less than or equal to 100%. If these criteria were met, the contig was further evaluated to determine if the alignments were in close proximity (within the region spanned by the contig plus 25 bp). If all these criteria were met, the contig was assumed to be a minor misassembly and reassigned as a presumptive correct assembly. Lastly, presumptive correct assemblies were checked for inversions by assessing the directionality of hits within the contig with respect to the hits in the reference genome. Scripts used to perform these analyses are provided (Supplementary Information 2).

### Statistics

Statistics were performed using a nonparametric Friedman test or rank-sum Wilcoxon tests (paired where applicable) in the R(v3.5) software package. ANOSIM correlations were performed on annotated matrices and NMDS plots were generated using the “Vegan” package (version 2.5–5) in the R software. Statistical significance was set at α = 0.05.

## Results

### Impact of sequencing technology and assembler on contig length distributions

Sample-matched short read Illumina and long-read nanopore sequencing runs were assembled for ten wastewater-derived samples using seven popular assemblers that leveraged differing assembly strategies for short, long, and hybrid (short and long) metagenomic reads. Only five out of ten long-read samples were able to be assembled by Canu within a five-day period on an institutional high-performance computing cluster with 32 cores and 493.59 GB of RAM. In contrast, all other assemblers successfully finished all samples in less than two days.

**Figure 1:**
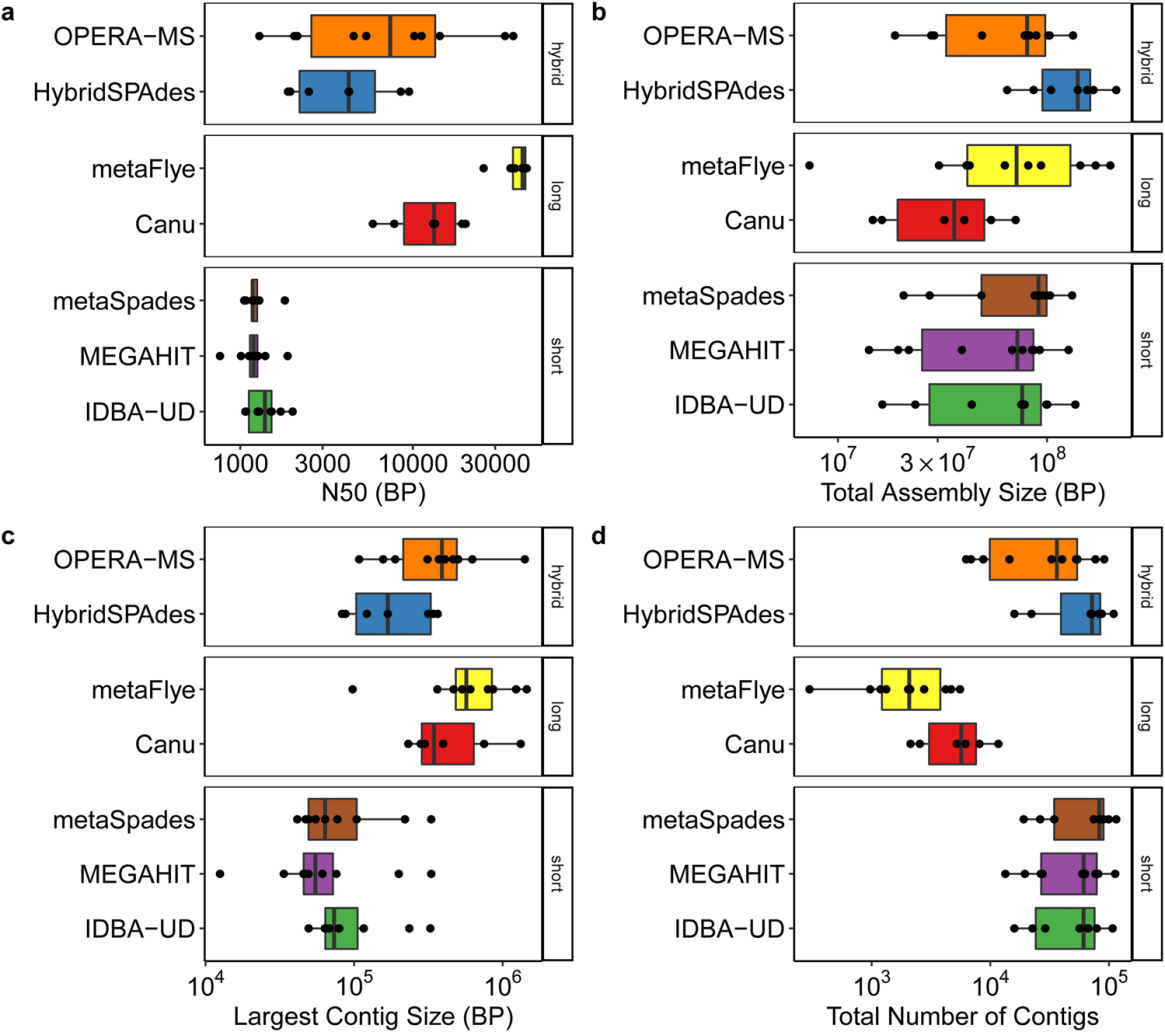
Descriptive assembly metrics for short, long, and hybrid read assemblies, where each point represents one of the ten samples assessed. The box inner line represents the median while the whiskers represent the 25th and 75th percentiles. (**a**) N50 of contig lengths. (**b**) Total assembly size in basepairs. (**c**) Largest contig size in basepairs. (**d**) Total number of contigs produced. This figure was generated using the ggplot2(v3.3.0) package in R(v3.5.0).

We first evaluated the sequenced metagenomes for common descriptive metrics (N50, total assembly size, maximum contig length, and total number of contigs produced), after filtering out contigs less than 500 bp (Fig. 1). N50 (the shortest contig length needed to capture 50% of the total assembly size) varied among short, long, and hybrid sequence assemblies (Fig. 1a), as did largest contig sizes (Fig. 1b), the total number of contigs (Fig. 1c), and the total assembly sizes (Fig. 1d) when comparing for each individual sample (e.g., USA-AS) (Friedman block test, respectively: p = 0.01, p < 0.0009, p < 0.00012, p = 0.0002).

IDBA-UD, metaSPAdes, and MEGAHIT produced similar N50s, but numbers of contigs produced by metaSPAdes differed (median contig number: 79,722), with a higher median contig number than MEGAHIT and IDBA-UD (median contig numbers: 61,204 and 61,284, respectively) (Fig. 1, Supplementary Table S5). Canu and metaFlye produced different N50 values (Supplementary Table S5), but the test was unbalanced because only six of ten samples could be assembled with Canu. Of the evaluated assemblers**,** metaFlye produced the longest, most contiguous assemblies and the smallest number of contigs overall (Fig. 1). Relative to short and long assemblies, the hybrid assemblers produced contig libraries with intermediate size distributions (Fig. 1). This is likely because HybridSPAdes and OPERA-MS first generate short read assemblies and then extend those assemblies using long-reads.

### Sequencing technology and assembler impact ARG contextualization

**Figure 2:**
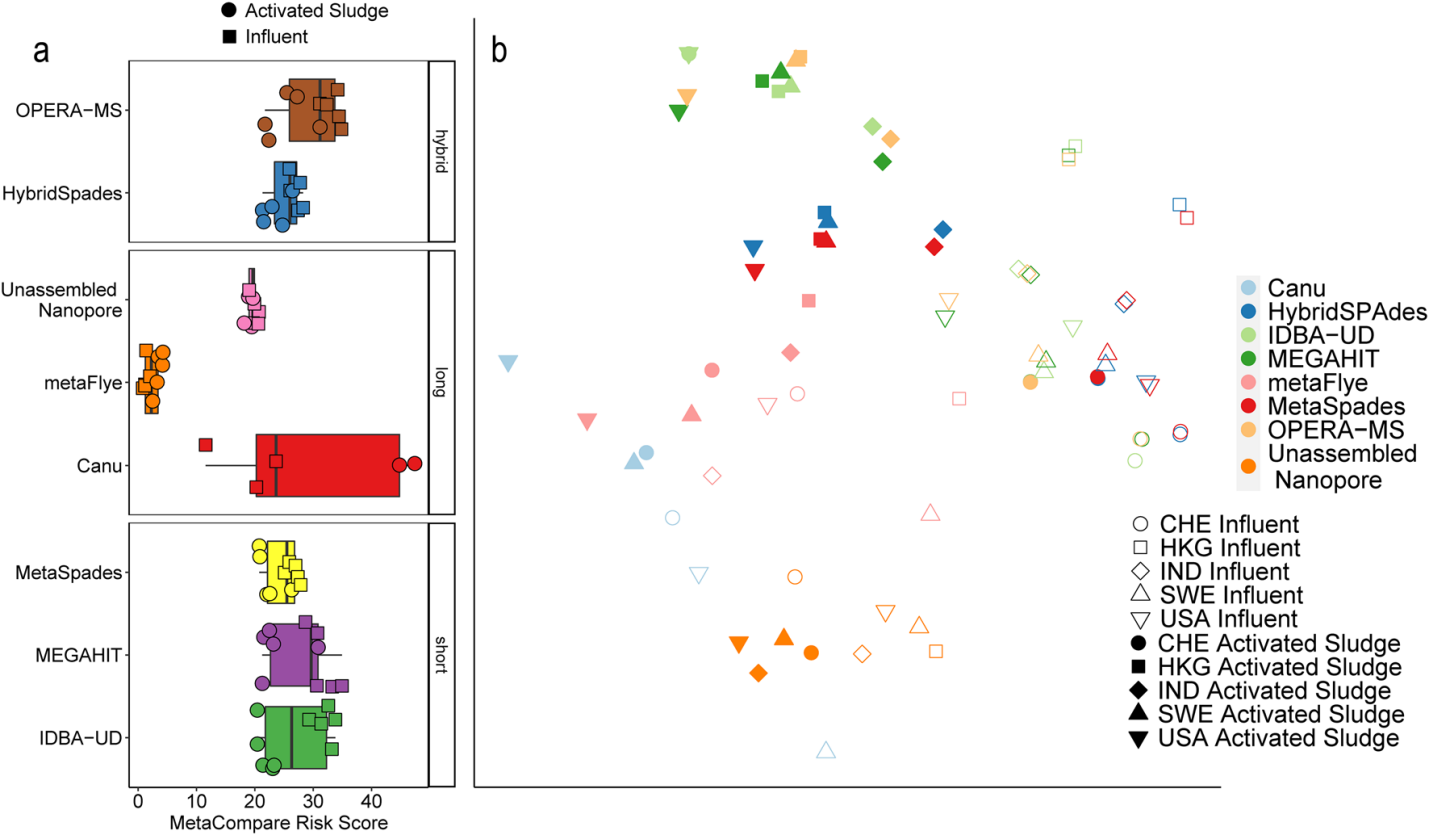
(**a**) Boxplot of MetaCompare risk scores by individual sample, where influent is represented as squares and activated sludge is represented as circles. The box inner line represents the median while the whiskers represent the 25th and 75th percentiles. (**b**) NMDS plot of annotated of ARGs, MGEs, and pathogen gene marker profiles underlying MetaCompare risk scores. Where *CHE* Switzerland, *HKG* Hong Kong, *IND* India, *SWE* Sweden, *USA* United States of America. Assembler type, treatment stage and sample were significant factors in system clustering (ANOSIM; R = 0.26, p = 0.001; R = 0.26, p = 0.001; R = 0.33, p = 0.001). This figure was generated using the ggplot2(v3.3.0) package in R(v3.5.0).

If the assemblers converge on a true underlying biological result, then it would be expected that they would produce similar profiles of co-occurrent genes. Antibiotic resistance is a key example of where information about gene co-occurrence is particularly valuable, as it can serve to inform with respect to whether ARGs are mobile (i.e., associated with an MGE) and/or putatively present in a pathogen. Consistency of assemblies in terms of relevant biological interpretation was thus assessed via MetaCompare^44^, which calculates a risk score based on the frequency of contig annotation with co-occurring ARGs, MGEs, and pathogen gene markers. Notably, different assemblers were found to produce distinct resistome risk scores for the same sample (Friedman, assembler by sample p < 0.0001). Additionally, the rankings of the risk scores changed between samples within assemblers as well, with Inf samples consistently ranking lower for long-read assemblers. This difference in assembler risk score was solely due to the long-read assemblers, possibly due to differences in the portions of the microbial community sequenced by the two respective technologies. It is unlikely that the error rate of nanopore sequences contributed substantially to the differences between sequencing technologies, as MetaCompare uses lenient criteria for annotating ARGs, MGEs, and pathogen markers (alignment length of 25 amino acids and 60% identity). Short read and hybrid assembly produced similar assessments of resistome risk (Fig. 2a), detected differences between samples (Supplementary Table S5), and generally ranked influent samples as having greater resistome risk than AS samples. On the other hand, long-read assembly predicted different relative resistome risk assessments (risk score and ranking of samples) and generally ranked AS samples as having less risk than influent (Fig. 2a). This demonstrates that choice of sequencing technology and assembly strategy may lead to different conclusions regarding relative resistome risk comparisons.

**Figure 3:**
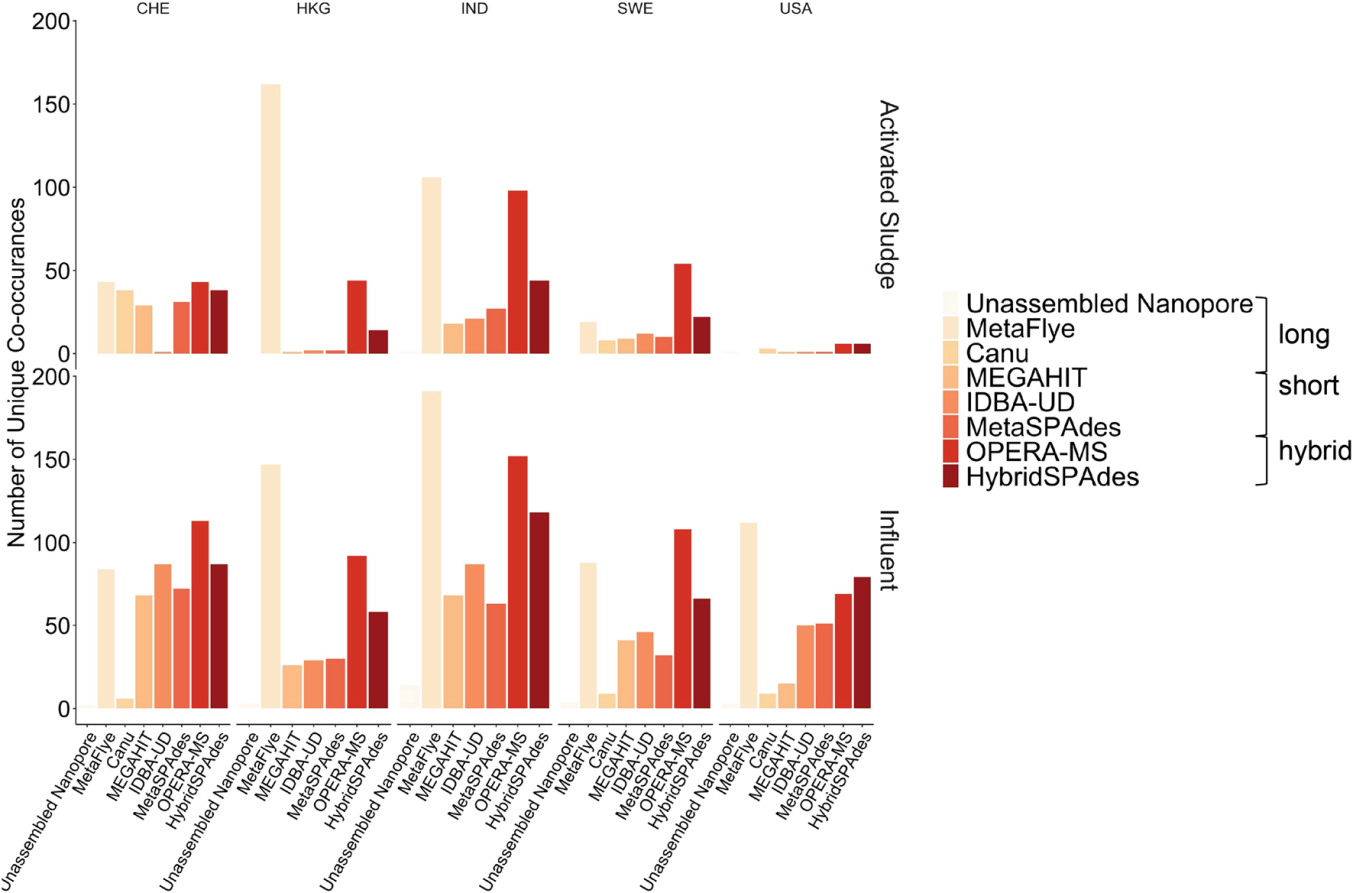
Number of unique co-occurrences of ARGs and MGEs identified among the samples (wastewater treatment plant- *CHE* Switzerland, *HKG* Hong Kong, *IND* India, *SWE* Sweden, *USA* United States of America; stage of treatment- Influent, Activated Sludge) as a function of assembly method. This figure was generated using the ggplot2(v3.3.0) package in R(v3.5.0).

We next investigated the complete profile of co-occurrent MGEs, ARGs, and pathogen gene markers underlying the MetaCompare risk score assessments (Fig. 2b). Both assembler and sample type displayed significant differences in co-occurrence profiles (i.e., AS or Inf) (ANOSIM; assembler: p = 0.001, R^2^ = 0.26, sample type: p = 0.001, R^2^ = 0.39; Fig. 2b). This was driven primarily by long-read assemblies and MinION reads (ANOSIM excluding long-read assemblies and MinION reads; assembler: p = 0.98, R^2^ = − 0.04, sample: p = 0.001, R = 0.64; Fig. 2b), highlighting the potential for the two sequencing technologies to yield different conclusions. Interestingly, while metaFlye produced the lowest risk scores of any assembler (Fig. 2a), it also predicted the greatest number of unique co-occurrences across all samples. Canu, on the other hand, predicted a similar number of co-occurrences as that observed in the unassembled MinION reads (Fig. 3). Examining how summary statistics correspond with ARG-MGE cooccurrences and risk scores, we observed significant positive correlations between total assembly size and both risk score and unique co-occurrences (Spearman: rho = 0.25, p = 0.04; rho = 0.60, p < 0.0001; respectively).

### Spiking an exogenous genome at various coverages to assess misassembly frequency

To aid in objectively evaluating the frequency of assembly error, we utilized a partial in silico experimental design wherein simulated short and long-reads from the *M. hydrocarbonoclasticus* ATCC 49,840 genome (NC_017067.1), an organism absent from the test samples (Supplemental Information Fig. S3-15), were combined with USA-AS and USA-Inf samples. Four short read (1×, 5×, 10×, 50×) and three long-read coverage (0.1×, 1×, and 3 × for USA-Inf or 5 × USA-AS). These coverages were selected to simulate low, medium, and high abundance scenarios, while the nanopore read sequence length distribution was simulated to correspond to that of the actual samples (Table S3). While the spiked-in nanopore read coverages are substantially lower, this was necessary to ensure that *M. hydrocarbonoclasticus* in the spiked-in reads did not exceed more than 20% of the total reads, which would represent an unlikely scenario in a true environmental sample (Table 1, Tables S3, S4). The spiked samples were then assembled with the seven different assemblers and the resulting assemblies were analyzed using a custom R algorithm to evaluate misassembly frequency.

**Figure 4:**
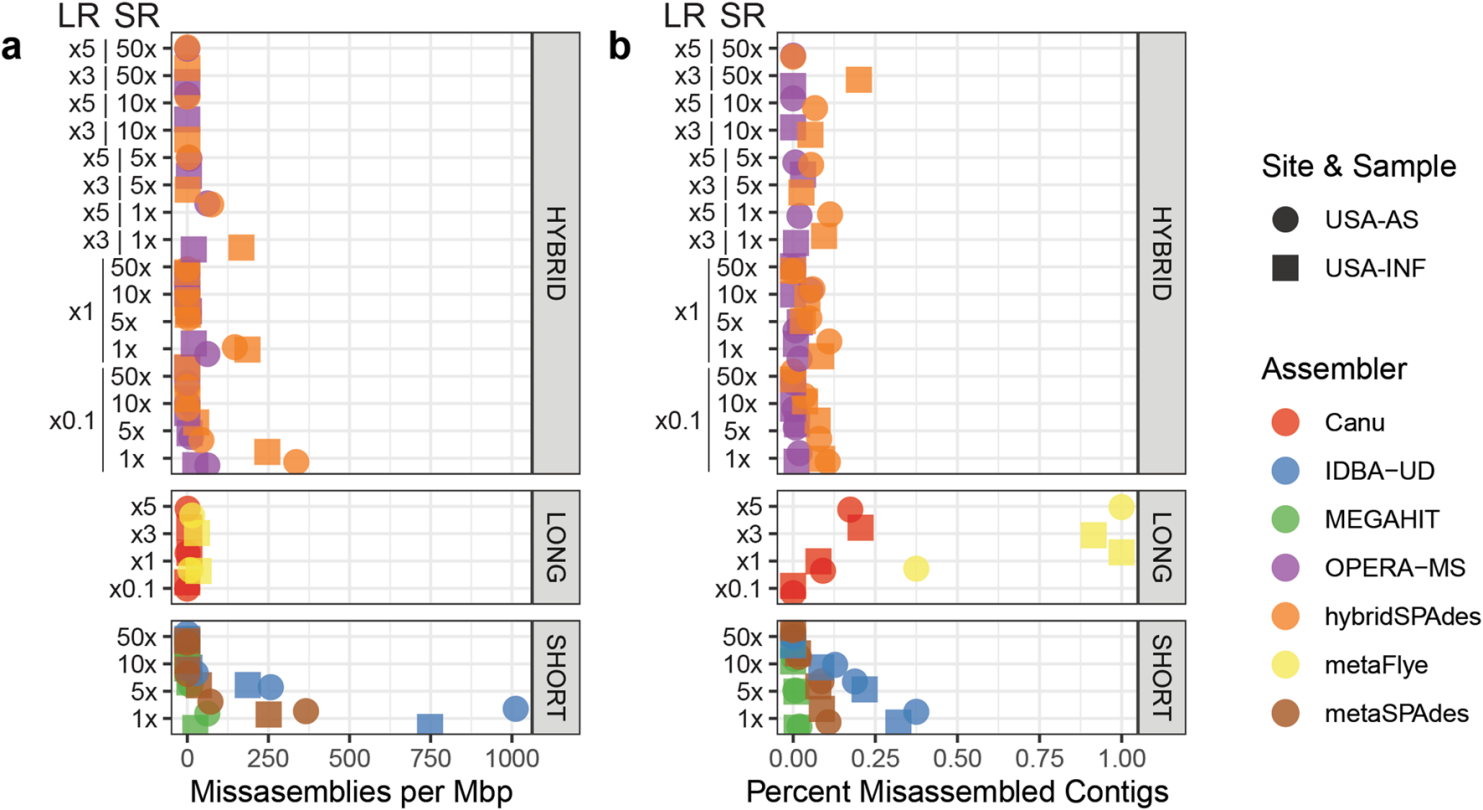
Assembler performance in assembling the in silico spiked genome of *Marinobacter hydrocarbonoclasticus* ATCC 49840 into two metagenomic samples (where circles represent USA-AS and squares represent USA-Inf). (**a**) Misassemblies per million basepairs within contigs aligning to the *M. hydrocarbonoclasticus* ATCC 8 genome. (**b**) Ratio of contigs with misassemblies to total contigs aligning to the *M. hydrocarbonoclasticus* genome. Y-axis indicates sequencing coverage, reported as long by short read coverages for hybrid assemblers. Hybrid assembly coverage is labeled with the long-read coverage by short-read coverage. This figure was generated using the ggplot2(v3.3.0) package in R(v3.5.0).

**Figure 5:**
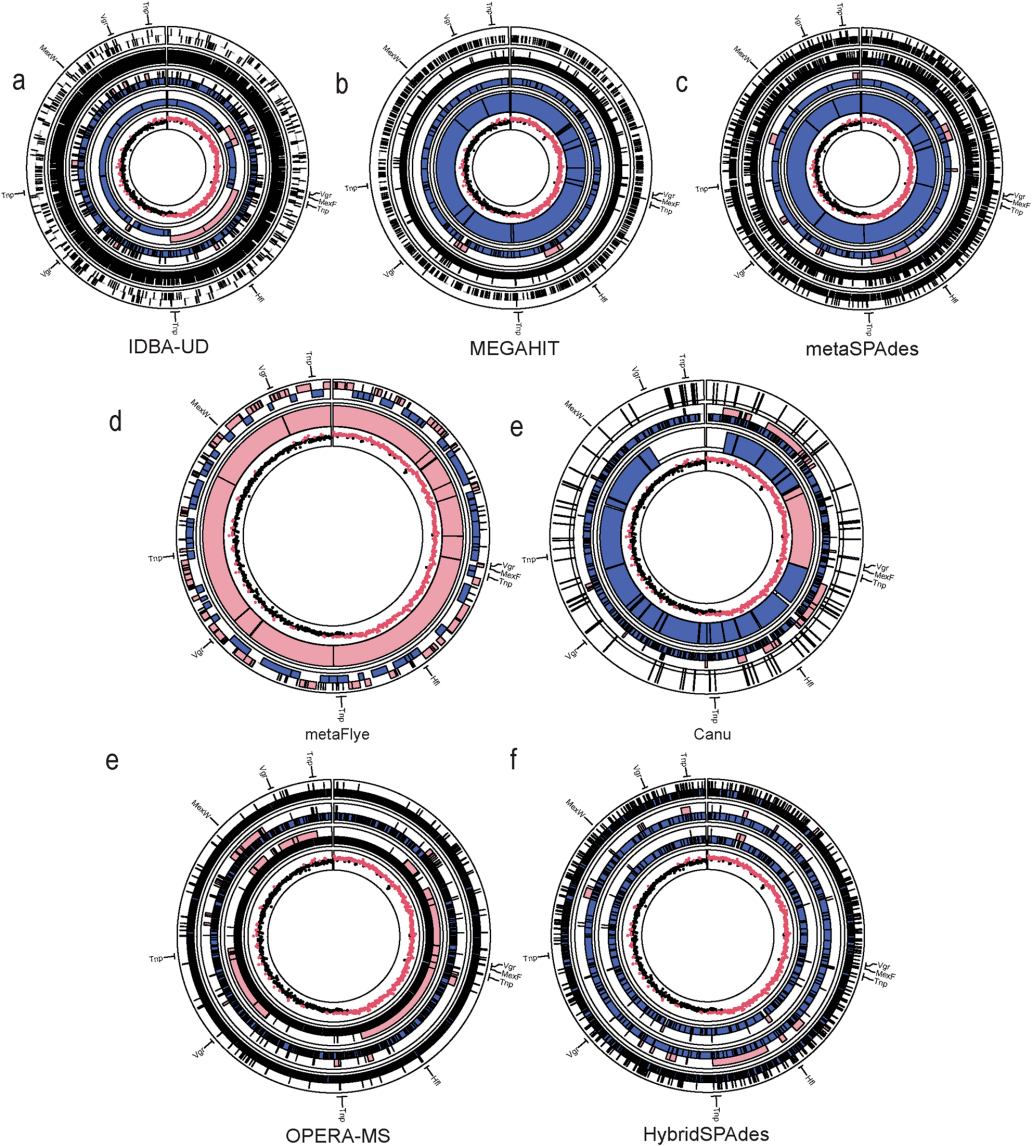
Circular depictions of the *Marinobacter hydrocarbonoclasticus* ATCC 49,840 genome with assembler type from USA-AS samples. (**a**) MEGAHIT with 1 × , 5 × , 10 × , 50×, alternating between misassemblies (pink) and correct assemblies (blue). (**b**) metaSPAdes with 1 × , 5 × , 10 × , 50×. (**c**) IDBA-UD with 1 × , 5 × , 10 × , 50×. (**d**) metaFlye with 0.1 × , 1 × , and 5 × coverage. (**d**) Canu with 1 × and 5 × coverage (no Flye contigs mapped at 0.1 × coverage). (**e**) Canu with 0.1 × , 1 × , and 5 × coverage. (**d**) Canu with 1 × and 5 × coverage (no Flye contigs mapped at 0.1 × coverage). (**f**) OPERA-MS with 10 × short-read and 0.1 × , 1 × , and 5 × long-read coverage. (**g**) HybridSpades with 10 × short-read and 0.1 × , 1 × , and 5 × long-read coverage. Labels are regions of the genome annotated with ARGs or MGE genes. Internal red/black dots are GC skew of 1000 basepair sections of the genome (red is positive GC skew while red–black is negative GC skew). This figure was generated using the Circlize(v0.4.11) package in R(v3.5.0).

When normalized to the total assembly size, the IDBA-UD assembly of metagenomes with the 1 × coverage spike resulted in the highest frequency of misassembly of *M. hydrocarbonoclasticus,* with nearly ten times more misassemblies than those produced by MEGAHIT and metaSPAdes at the same coverage (Fig. 4). Interestingly, spiking 5 × coverage of the reference genome into short reads resulted in the assembly of a nearly complete, but discontiguous, reference genome by the short read assemblers (Fig. 5a–c). However, there were still a substantial number of misassemblies (Figs. 4a, 5a–c). This suggests that, while the entire genome could essentially be recovered from metagenomes with the 5 × spike, there was additionally a high frequency of incorrect contigs incurred, likely because of assembly of reads from different genomes into chimeric contigs.

Canu and metaFlye produced contiguous, mostly accurate, assemblies under conditions of higher coverage (Fig. 4a,b). metaFlye performed well at higher coverages. For instance, the ostensibly poor performance apparent in Fig. 5d was due to two contigs, one of which was a likely interspecies translocation (Supplementary Information Fig. S15A) and the other was a small indel within a contig that extended the entire length of the genome (Fig. S15B). At 0.1 × coverage, no metaFlye contigs could be confidently mapped to the reference genome, as they did not meet the background filtering step criteria (> 95% identity and alignment length > 210 bp). This could relate to the high error rate associated with MinION sequencing. Canu, however, was able to produce 13 correctly assembled contigs for USA-Inf and 69 for USA-AS, which all mapped to the reference genome at 0.1×, likely due to its read correction step. Nonetheless, given the extensive computational time and power required to run Canu, our results indicate that we did not arrive at an optimal approach for assembly of nanopore reads from complex environmental metagenomes among the tested methods.

Relative to the long- and short- read assemblies, the hybrid assemblers performed well. Among hybrid assemblers, low short-read coverage samples (1×) produced the most frequent assembly errors relative to assembly size, with more errors produced by samples with higher MinION coverage.

**Figure 6:**
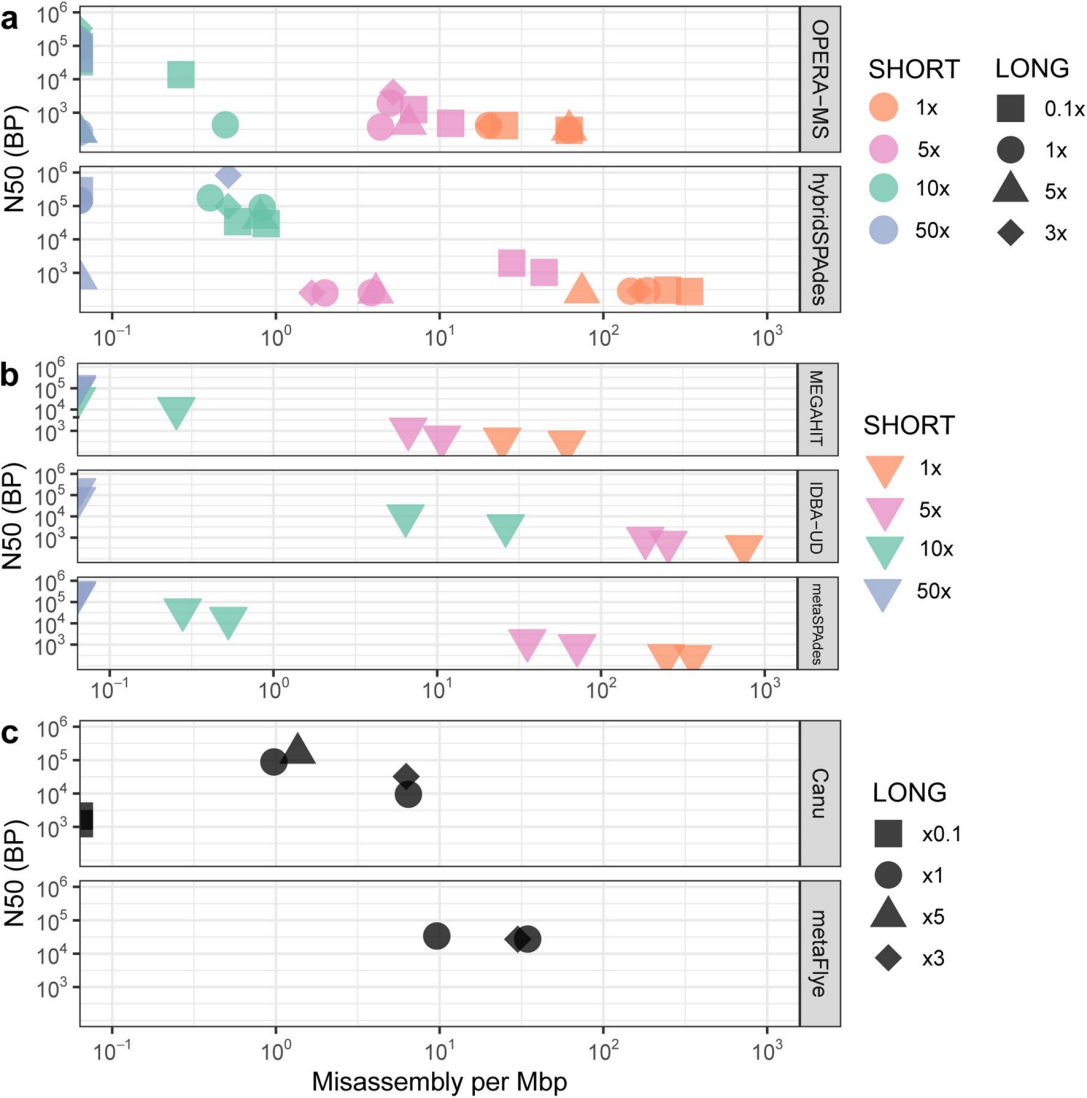
Comparison of Misassemblies per Mbp as determined through our contig classifier (SI 2) compared to assembly contiguity (N50) for (**a**) hybrid assemblers, (**b**) short-read assemblers and (**c**) long-read assemblers. This figure was generated using the ggplot2(v3.3.0) package in R(v3.5.0).

Finally, it is noted that hybrid assembly boosted contiguity (here measured as N50) and reduced assembly errors in the in silico spike experiment, particularly at intermediate short-read coverages (5 × –10 ×) (Figs. 5, 6). However, contiguity and accuracy were increased with hybrid assembly with increasing coverage in the short, but not the long, reads (Fig. 6). This emphasizes that increased long-read sequencing depth will not compensate for lower short-read sequencing depth for the hybrid assemblers evaluated for this study.

## Discussion

Here we evaluated the impact of hybrid-, long-, and short-read assembly methodologies on the quality and accuracy of assemblies derived from complex environmental metagenomes. The implications of the resulting assemblies for biological interpretation were assessed by examining the contextualization of ARGs as an exemplar.

metaFlye produced the greatest number of contigs containing co-occurring ARGs and MGEs across most samples (Fig. 3), but produced the lowest resistome risk scores. Additionally, metaFlye produced differing rankings of risk scores than other assemblers, though, overall, the trend of Inf producing a lower risk score than AS was consistent with Canu. It is expected that AS would produce a lower risk score than Inf, because it is widely known that pathogens decrease, as reduction of human pathogens is one of the primary functions of activated sludge (Wery et al. 2008). Further, recent studies have also shown that there tends to be a net reduction in MGEs (Che et al. 2019). Examining the MetaCompare data more closely (Supplementary 1 Table S6), it is apparent that metaFlye yielded the smallest proportion of ARG-MGE-pathogen annotated contigs. This suggests that the large number of unique co-occurrences observed across samples (Fig. 3) are likely due to a small number of contigs with both ARG and MGE annotations. Furthermore, the high frequency of assembly error produced by metaFlye in the in silico spike experiment is concerning (Figs. 4, 5d), thus casting doubt on the large number of unique co-occurrences of MGEs and ARGs resulting from this assembly approach (Fig. 3), which could be the result of chimeric contigs producing false positive associations. MetaFlye performed well at higher coverages, but it is possible that implementing error correction strategies, such as those that target indels could lead alter biologically relevant results^54^. Canu produced similar numbers of co-occurrences relative to the unassembled MinION reads. When considering this and the greater accuracy of Canu (Fig. 5c), it is likely that the frequency of co-occurrences was more accurately reflected the microbial community represented within the MinION reads. These results highlight that descriptive metrics (Fig. 1) can be a deceptive indicator of assembly quality because, while metaFlye produced the largest most contiguous assembly, there was also likely a high frequency of assembly error that would be undetected without further scrutiny.

Comparing to previous studies, Latorre-Perez et al*.* 2020 found that available MinION assembly algorithms were able to accurately assemble simple metagenomes of mock microbial communities that were subjected to deep sequencing (14–16 giga basepairs per sample that were subsampled 3 and 6 Gbp)^55^, the present study illustrates that there are still significant shortcomings in the application of these pipelines to more complex environmental metagenomes. While it is possible that the decreased error rate^56^ provided by PacBio HiFi sequencing might improve assembly, we suspect that sequencing depth is a larger driver of assembly error rate. Additionally, we note that we did not explore the possibility of error correction in the long-read assemblies. Past work by Arumugam et al.^54^ has shown, in less diverse communities, that frame shift errors present in long-read assemblies can affect translation. However, recent work by Arango-Argoty et al.^57^, which examined ARG detection in complex environmental samples, did not find frame shift errors to be problematic and further demonstrated that the number of ARGs detected did not increase with additional error correction. Furthermore, Canu performs a read polishing step as a part of its assembly pipeline.

We observed that different types of errors are prevalent at different sequencing depths, i.e., at different coverages for a given genome within a sample. For instance, at 5 × coverage of *M. hydrocarbonoclasticus* spiked into the short-read metagenomes, there was a high frequency of misassembly associated with the incorporation of unrelated reads into contigs (Figs. 4a, 5a,b). This follows prior observations that species abundance in microbial communities follows a power law^19,58^, wherein low abundance species are present at similar levels, leading to difficulty in distinguishing unrelated reads with overlapping *k-*mers on the basis of coverage. On the other hand, at higher coverages (i.e., 10 × and 50 × for short reads, and 3 × and 5 × for MinION reads) across all assemblers, the contigs that mapped to the reference genomes showed a tendency to produce inversions and indels at a greater frequency than chimeras (Fig. 5). This suggests that contigs with higher coverage are less likely to represent false-positive associations. These results suggest that one strategy to ensure validity of assembly-based resistome analyses would be to exclude contigs below a given depth, e.g., 10×, as these are more likely to be chimeric.

When designing a metagenomic sequencing experiment, one must weigh many variables, including research objectives and cost. While, in the present work, we do not directly evaluate the important consideration of cost, we found that hybrid assembly boosted contiguity and accuracy at coverages that are relevant to complex environmental metagenomes (Fig. 6a,b). OPERA-MS and HybridSPAdes performed comparably, but both yielded frequent misassemblies, which interestingly often occurred at different regions of the reference genome (Fig. 5e,f). Therefore, one strategy to minimize the incorrect inferences drawn by assembly of metagenomes might be to instead leverage the consensus of multiple tools. Last, because environmental metagenomes remain undersequenced relative to other targets, such as the human gut, the reference sequence-based binning strategy of OPERA-MS may make it intrinsically better suited towards more well-archived environments.

## Conclusions

This work presents the first critical assessment of methodologies for short-, long-, and hybrid- assembly of metagenomes derived from complex environmental samples. In sum, the present study supports hybrid assembly as a valuable technique for boosting contiguity and increasing accuracy of metagenome assembly, but also emphasizes the need for adequate short-read sequencing depth to harness the full potential of the approach. The findings of this study provide key information towards informing a framework for guiding selection of sequencing platform(s), depths, and assembly methodologies for complex environmental samples.

## Supplementary Information

Supplementary Information 1.

Supplementary Information 2.

## Data Availability

The assembled and raw reads used for this study are available on NCBI SRA (https://www.ncbi.nlm.nih.gov/bioproject/PRJNA527877). Supplementary Table 1 delineates which SRA ID corresponds to which sample. Code used in this study is available in SI II.
