# Supplementary material for: Critical evaluation of short, long, and hybrid assembly for contextual analysis of antibiotic resistance genes in complex environmental metagenomes
Source: Sci Rep. 2021 Feb 12;11:3753. doi: 10.1038/s41598-021-83081-8 (PMC7881036; doi:10.1038/s41598-021-83081-8)
Supplement: Supplementary file 1 — Supplementary Information 1. [file 41598_2021_83081_MOESM1_ESM.pdf]

Supplemental Information 1:

Critical evaluation of short, long, and hybrid assembly for contextual analysis of antibiotic resistance genes in complex environmental metagenomes

Authors: *Connor Brown[1]*, Ishi Keenum[2]*, Dongjuan Dai[2], Liqing Zhang[1], Peter Vikesland[2], Amy Pruden[2]*

[1]Virginia Tech, Genetics, Bioinformatics, and Computational Biology, Blacksburg, VA 24060
[2]Virginia Tech, Department of Civil & Environmental Engineering, Blacksburg, VA 24060

*These authors contributed equally to the work
Corresponding authors: Amy Pruden and Liqing Zhang

Table S1: NCBI SRA Correlations in NCBI BioProject PRJNA527877

Sample ID	Assembler	NCBI BioSample	NCBI SRA
USA-1-influent	IDBA-UD	SAMN16129134	SRR12664620
USA-1-influent	Megahit	SAMN16129135	SRR12664619
USA-1-influent	Canu	SAMN16129136	SRR12664608
USA-1-influent	OPERA-MS	SAMN16129137	SRR12664597
USA-1-influent	HybridSpades	SAMN16129138	SRR12664586
USA-1-influent	Flye	SAMN16129139	SRR12664575
USA-1-influent	Nanopore	SAMN16129140	SRR12664564
USA-1-influent	metaSPAdes	SAMN16816579	SRR13105837
USA-1-activated sludge	IDBA-UD	SAMN16129141	SRR12664557
USA-1-activated sludge	Megahit	SAMN16129142	SRR12664556
USA-1-activated sludge	Canu	SAMN16129143	SRR12664555
USA-1-activated sludge	OPERA-MS	SAMN16129144	SRR12664618
USA-1-activated sludge	HybridSpades	SAMN16129145	SRR12664617
USA-1-activated sludge	Flye	SAMN16129146	SRR12664616
USA-1-activated sludge	Nanopore	SAMN16129147	SRR12664615
USA-1-activated sludge	metaSPAdes	SAMN16816580	SRR13105836
SWE-1-influent	IDBA-UD	SAMN16129148	SRR12664614
SWE-1-influent	Megahit	SAMN16129149	SRR12664613
SWE-1-influent	Canu	SAMN16129150	SRR12664612
SWE-1-influent	OPERA-MS	SAMN16129151	SRR12664611
SWE-1-influent	HybridSpades	SAMN16129152	SRR12664610
SWE-1-influent	Flye	SAMN16129153	SRR12664609
SWE-1-influent	Nanopore	SAMN16129154	SRR12664607
SWE-1-influent	metaSPAdes	SAMN16816581	SRR13105835
SWE-1-activated sludge	IDBA-UD	SAMN16129155	SRR12664606
SWE-1-activated sludge	Megahit	SAMN16129156	SRR12664605
SWE-1-activated sludge	Canu	SAMN16129157	SRR12664604
SWE1-activated sludge	OPERA-MS	SAMN16129158	SRR12664603
SWE-1-activated sludge	HybridSpades	SAMN16129159	SRR12664602
SWE-1-activated sludge	Flye	SAMN16129160	SRR12664601
SWE-1-activated sludge	Nanopore	SAMN16129161	SRR12664600
SWE-1-activated sludge	metaSPAdes	SAMN16816582	SRR13105834
CHE-1-influent	IDBA-UD	SAMN16129162	SRR12664599
CHE-1-influent	Megahit	SAMN16129163	SRR12664598
CHE-1-influent	Canu	SAMN16129164	SRR12664596
CHE-1-influent	OPERA-MS	SAMN16129165	SRR12664595
CHE-1-influent	HybridSpades	SAMN16129166	SRR12664594

CHE-1-influent	Flye	SAMN16129167	SRR12664593
CHE-1-influent	Nanopore	SAMN16129168	SRR12664592
CHE-1-influent	metaSPAdes	SAMN16816583	SRR13105833
CHE-1-activated sludge	IDBA-UD	SAMN16129169	SRR12664591
CHE-1-activated sludge	Megahit	SAMN16129170	SRR12664590
CHE-1-activated sludge	Canu	SAMN16129171	SRR12664589
CHE1-activated sludge	OPERA-MS	SAMN16129172	SRR12664588
CHE-1-activated sludge	HybridSpades	SAMN16129173	SRR12664587
CHE-1-activated sludge	Flye	SAMN16129174	SRR12664585
CHE-1-activated sludge	Nanopore	SAMN16129175	SRR12664584
CHE-1-activated sludge	metaSPAdes	SAMN16816584	SRR13105832
IND-1-influent	IDBA-UD	SAMN16129176	SRR12664583
IND-1-influent	Megahit	SAMN16129177	SRR12664582
IND-1-influent	OPERA-MS	SAMN16129178	SRR12664581
IND-1-influent	HybridSpades	SAMN16129179	SRR12664580
IND-1-influent	Flye	SAMN16129180	SRR12664579
IND-1-influent	Nanopore	SAMN16129181	SRR12664578
IND-1-influent	metaSPAdes	SAMN16816585	SRR13105831
IND-1-activated sludge	IDBA-UD	SAMN16129182	SRR12664577
IND-1-activated sludge	Megahit	SAMN16129183	SRR12664576
IND1-activated sludge	OPERA-MS	SAMN16129184	SRR12664574
IND-1-activated sludge	HybridSpades	SAMN16129185	SRR12664573
IND-1-activated sludge	Flye	SAMN16129186	SRR12664572
IND-1-activated sludge	Nanopore	SAMN16129187	SRR12664571
IND-1-activated sludge	metaSPAdes	SAMN16816586	SRR13105830
HKG-1-influent	IDBA-UD	SAMN16129188	SRR12664570
HKG-1-influent	Megahit	SAMN16129189	SRR12664569
HKG-1-influent	OPERA-MS	SAMN16129190	SRR12664568
HKG-1-influent	HybridSpades	SAMN16129191	SRR12664567
HKG-1-influent	Flye	SAMN16129192	SRR12664566
HKG-1-influent	Nanopore	SAMN16129193	SRR12664565
HKG-1-influent	metaSPAdes	SAMN16816587	SRR13105829
HKG-1-activated sludge	IDBA-UD	SAMN16129194	SRR12664563
HKG-1-activated sludge	Megahit	SAMN16129195	SRR12664562
HKG1-activated sludge	OPERA-MS	SAMN16129196	SRR12664561
HKG-1-activated sludge	HybridSpades	SAMN16129197	SRR12664560
HKG-1-activated sludge	Flye	SAMN16129198	SRR12664559
HKG-1-activated sludge	Nanopore	SAMN16129199	SRR12664558
HKG-1-activated sludge	metaSPAdes	SAMN16816588	SRR13105828

Table S2: Simulated short read characteristics.

Platform Simulated	Sample	Targeted Genome Depth	Simulated Paired-Reads
HiSeq (x100bp)	USA-Influent	1x	1.99E+05
HiSeq (x100bp)	USA-Influent	5x	9.97E+04
HiSeq (x100bp)	USA-Influent	10x	1.99E+05
HiSeq (x100bp)	USA-Influent	50x	9.97E+05
NextSeq (x75bp)	USA-AS	1x	2.66E+04
NextSeq (x75bp)	USA-AS	5x	1.33E+05
NextSeq (x75bp)	USA-AS	10x	2.66E+05
NextSeq (x75bp)	USA-AS	50x	1.33E+06

Table S3: Read length characteristics used in simulation of Nanopore reads.

USA-AS	Max Read Size	52276 bp
	Min Read Size	130 bp
	Median	2800 bp
	log(SD)	0.49
USA-Influent	Max Read Size	27885 bp
	Min Read Size	207 bp
	Median	1105 bp
	log(SD)	0.34

Table S4: NanoSIMs Reads Output

Coverage	Sample	Aligned BP	Unaligned BP	Aligned Reads	Unaligned Reads	total
x0.1	USA INF	5.88E+06	3.96E+05	4.57E+03	3.68E+02	4.94E+03
x1	USA INF	6.29E+07	4.28E+06	4.57E+04	3.69E+03	4.94E+04
x3	USA INF	1.88E+08	1.28E+07	1.37E+05	1.11E+04	1.48E+05
x0.1	USA AS	6.15E+06	5.58E+05	1.85E+03	1.49E+02	2.00E+03
x1	USA AS	6.13E+07	4.81E+06	1.85E+04	1.50E+03	2.00E+04
x5	USA AS	3.07E+08	2.27E+07	9.28E+04	7.5E+03	1.00E+05

Table S5: Statistics on Summary Metrics

Comparing: group1 (n compared;median)	To: group1 (n compared;median)	Based on metric:	P value	Statistical Test
MEGAHIT, IDBA-UD, metaSPAdes		N50	0.20	Friedman
MEGAHIT, IDBA-UD, metaSPAdes		# contigs	0.0006	Friedman
OPERA-MS (10; 371.5 kbp)		HybridSpades (10; 125.0 kbp)	Largest Contig	0.01
OPERA-MS (10; 78.2 Mbp)	HybridSpades (10; 118.0 Mbp)	Total Assembly Size	0.00056	Paired Wilcox
OPERA-MS (10; 5.3 kbp)	HybridSpades (10; 3.4 kbp)	N50	0.012	Paired Wilcoxon
Canu (5; 10.6 kbp)	Flye (10; 43.3 kbp)	N50	0.002	Paired Wilcoxon

Hybrid assemblers- Opera-MS & HybridSpades (26.85)	Short read assemblers – megahit, metaSPAdes, IDBA-UD (26.1)	Risk Score	0.42	Wilcoxon
Hybrid & short assemblers- Opera-MS, HybridSpades, megahit, metaSPAdes, IDBA (26.4)	SMRT assemblers - Canu & Flye (4.2)	Risk Score	<0.0001	Wilcoxon

Table S6: Breakdown of MetaCompare Risk Scores

Sample	Assembler	Stage	Risk score	nContigs	nARG	nMGE	nPAT	nARG &MGE	nARG &MGE &PAT
USA	Canu	inf	11.6	2125	54	30	107	5	1
CHE	Canu	inf	20.3	2548	51	27	56	3	0
SWE	Canu	inf	23.6	5222	100	39	245	4	3
SWE	Canu	as	44.8	6194	88	27	37	5	1
CHE	Canu	as	47.4	11701	90	23	46	4	2
CHE	FMLRC	inf	17.6	550203	4	1	2	0	0
USA	FMLRC	as	17.9	547303	71	8	11	0	0
CHE	FMLRC	as	17.9	759368	101	13	20	0	0
HKG	FMLRC	as	18.1	2999536	759	133	248	2	1
IND	FMLRC	as	18.2	1503839	428	46	32	1	0
HKG	FMLRC	inf	19	2343436	1508	252	874	2	0
SWE	FMLRC	as	19.7	547347	502	70	57	0	0
IND	FMLRC	inf	19.9	833396	840	98	364	9	2
SWE	FMLRC	inf	20.6	1068885	1382	268	705	5	0
USA	FMLRC	inf	20.7	718617	954	182	488	3	1
IND	HybridSpades	as	21.3	604077	923	1228	442	15	4
USA	HybridSpades	as	21.5	108095	170	248	106	4	2
SWE	HybridSpades	as	22.9	145425	298	568	146	10	2
HKG	HybridSpades	as	24.7	98906	262	499	230	7	1
IND	HybridSpades	inf	25.9	631270	1824	2108	9362	82	30
HKG	HybridSpades	inf	26.1	500791	1517	2095	17257	40	14
CHE	HybridSpades	as	26.5	671406	2148	2896	6168	40	8

SWE	HybridSpade s	inf	27.4	550013	1895	2817	6049	41	11
CHE	HybridSpade s	inf	27.8	689277	2407	4119	1365 7	70	24
USA	HybridSpade s	inf	28.3	597391	2185	2871	1047 0	58	16
USA	IDBA-UD	as	20.4	37785	44	187	30	1	1
CHE	IDBA-UD	as	20.4	37785	44	188	30	1	1
IND	IDBA-UD	as	21.4	160174	241	903	138	8	4
SWE	IDBA-UD	as	23.1	60632	123	486	57	6	2
HKG	IDBA-UD	as	23.3	49824	111	410	85	2	0
HKG	IDBA-UD	inf	29.3	153507	555	1848	6157	33	18
SWE	IDBA-UD	inf	31.4	163656	683	2819	2332	40	13
USA	IDBA-UD	inf	32.6	173704	766	2955	4106	48	15
CHE	IDBA-UD	inf	33.2	215914	982	4221	6612	60	20
IND	IDBA-UD	inf	33.8	145306	584	1732	2696	81	30
IND	MEGAHIT	as	21.3	205867	305	970	232	8	4
USA	MEGAHIT	as	21.5	33636	53	176	34	1	1
SWE	MEGAHIT	as	22.5	58172	110	456	50	4	1
HKG	MEGAHIT	as	23.2	45411	99	370	62	2	0
HKG	MEGAHIT	inf	28.7	161238	582	1708	5969	25	12
SWE	MEGAHIT	inf	30.6	193039	809	2601	2809	28	10
USA	MEGAHIT	inf	30.7	98668	414	797	1585	16	5
CHE	MEGAHIT	as	30.8	203008	863	2735	1945	32	8
CHE	MEGAHIT	inf	33.3	270763	1291	3920	7935	54	16
IND	MEGAHIT	inf	34.9	163612	719	1756	3224	82	30
IND	MetaFlye	inf	0.8	299	41	32	87	10	8
SWE	MetaFlye	inf	1.2	2113	196	195	384	37	18
CHE	MetaFlye	inf	1.2	1328	122	146	147	27	11
USA	MetaFlye	inf	1.4	971	77	93	113	24	13
HKG	MetaFlye	inf	2.0	5555	332	346	352	54	23
SWE	MetaFlye	as	2.5	2028	105	58	60	8	4
CHE	MetaFlye	as	3.3	2766	123	91	68	16	4
HKG	MetaFlye	as	3.5	4669	204	221	132	37	13
IND	MetaFlye	as	4.2	4205	169	153	124	33	17
USA	MetaFlye	as	4.3	1188	44	19	22	0	0
IND	MetaSpades	as	20.8	773030	1034	1133	483	10	3
USA	MetaSpades	as	20.9	140405	194	236	130	1	1
SWE	MetaSpades	as	22	195680	346	551	158	8	3

HKG	MetaSpades	as	22.6	144007	285	472	242	4	1
HKG	MetaSpades	inf	25.1	621300	1735	2001	20029	28	10
IND	MetaSpades	inf	25.9	669015	1952	2078	10084	75	25
CHE	MetaSpades	as	26.3	685840	2171	2889	6190	38	8
SWE	MetaSpades	inf	27	629711	2115	2758	7100	31	12
CHE	MetaSpades	inf	27.4	738633	2516	4071	14271	66	20
USA	MetaSpades	inf	27.9	649361	2327	2810	12016	45	16
IND	OPERA-MS	as	21.7	169322	265	799	169	19	4
USA	OPERA-MS	as	22.4	26503	46	177	37	4	1
SWE	OPERA-MS	as	25.5	40268	94	431	47	16	4
HKG	OPERA-MS	as	27.3	32736	100	383	70	10	1
HKG	OPERA-MS	inf	31.1	134573	548	1556	5521	33	14
CHE	OPERA-MS	as	31.2	200285	861	2731	1919	36	8
SWE	OPERA-MS	inf	32.3	164563	715	2390	2052	45	15
CHE	OPERA-MS	inf	34.2	249144	1198	3705	7670	73	17
USA	OPERA-MS	inf	34.4	85623	394	815	1496	34	9
IND	OPERA-MS	inf	34.8	154915	677	1683	3065	79	28

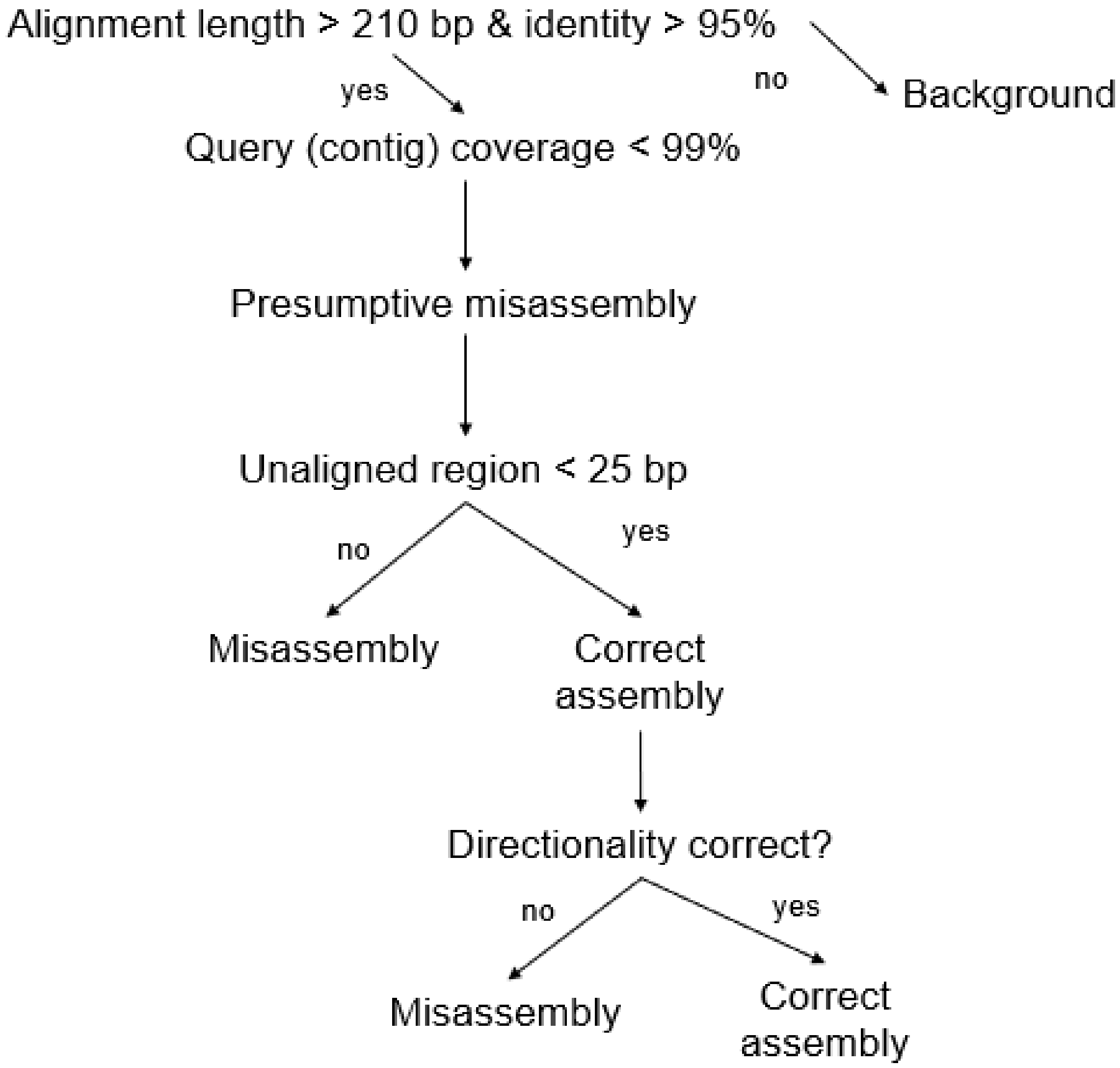

Figure S1. Logic flow of the R function to detect misassemblies.

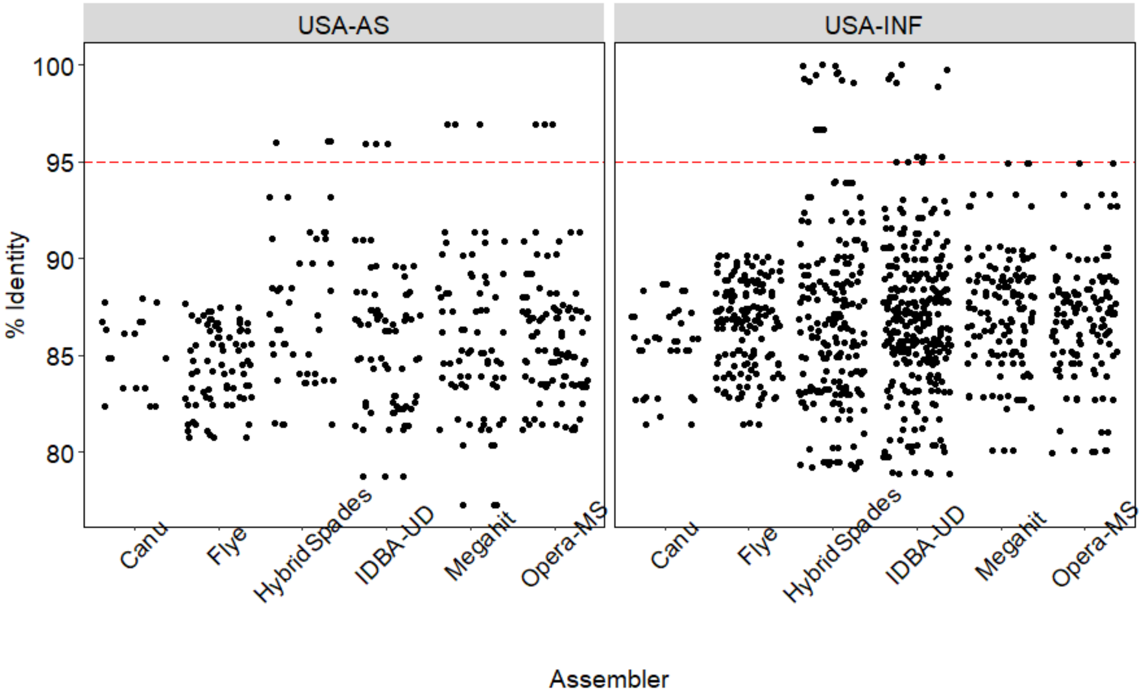

Figure S2. Distribution of Nucmer alignments produced from non-spiked assemblies. This data was used as criteria to establish an identity and alignment cut-off of 95% and 210 bp to filter out background. These criteria eliminated greater than 97% of the background hits from non-spiked samples.

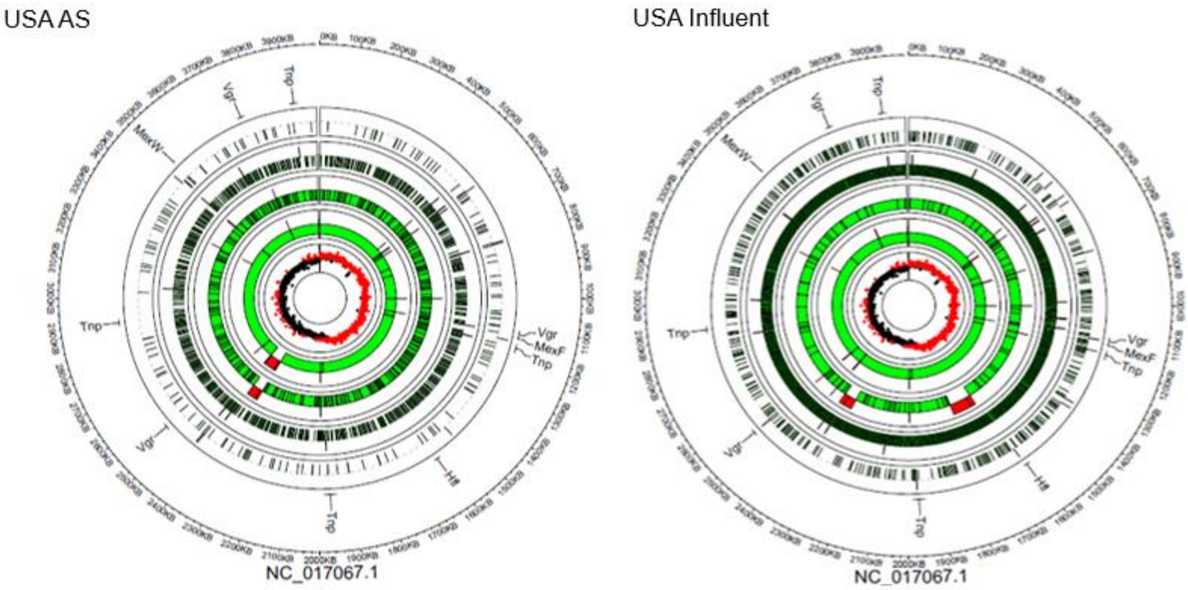

Figure S3. Hits to reference genome from Megahit assemblies of the *in silico* spiked metagenomes. From outside towards the inside: 1x (misassembly), 1x (correct assembly); 5x, 10x, 50x. Green indicates a contig that was correctly assembled. Red indicates a contig with a misassembly. Made with R(v3.5.0) and Circlize(v0.4.11).

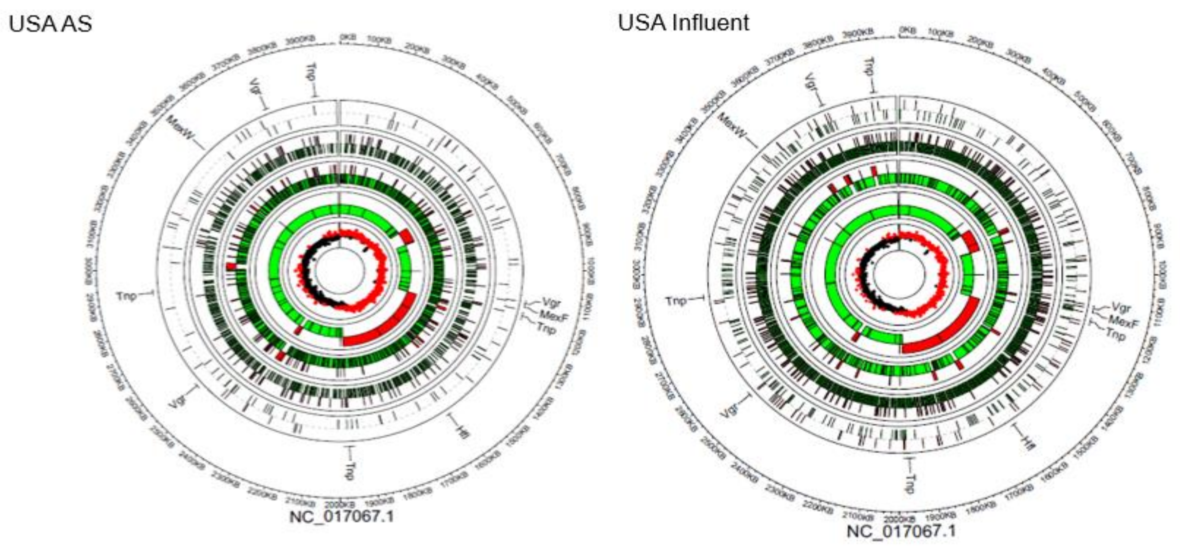

Figure S4. Hits to reference genome from IDBA-UD assemblies of the *in silico* spiked metagenomes. From outside towards the inside: 1x (misassembly), 1x (correct assembly); 5x, 10x, 50x. Green indicates a contig that was correctly assembled. Red indicates a contig with a misassembly. Made with R(v3.5.0) and Circlize(v0.4.11).

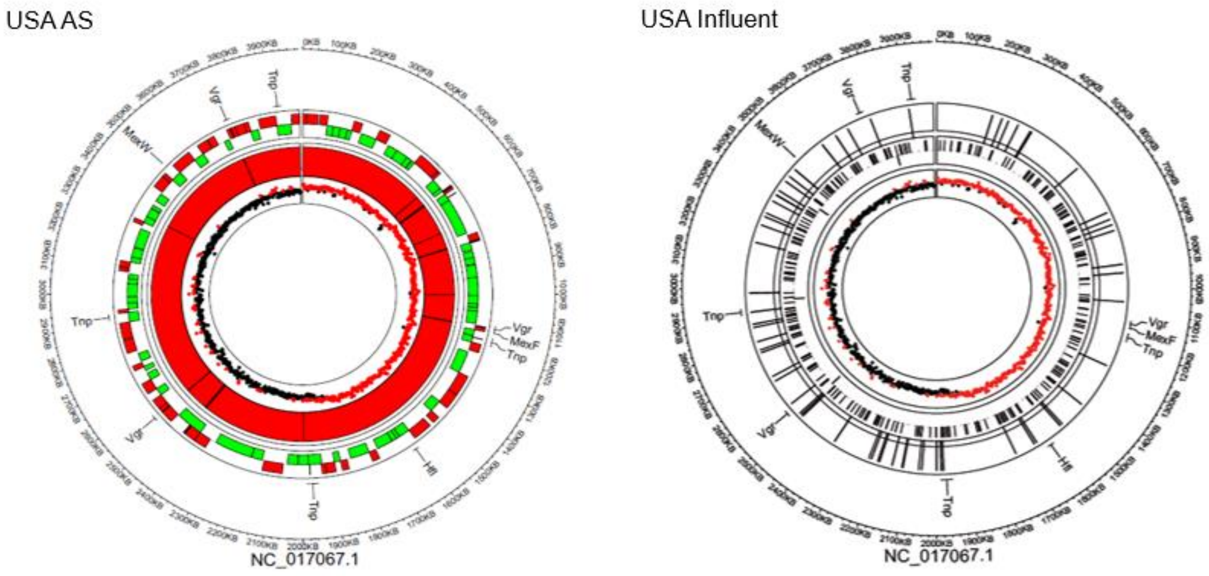

Figure S5. Hits to reference genome from metaFlye assemblies of the *in silico* spiked metagenomes. From outside towards the inside:1x, 3x (influent) or 5x (AS). Green indicates a contig that was correctly assembled. Red indicates a contig with a misassembly. No x0.1 spiked sample assembled any contigs that passed background screening. Made with R(v3.5.0) and Circlize(v0.4.11).

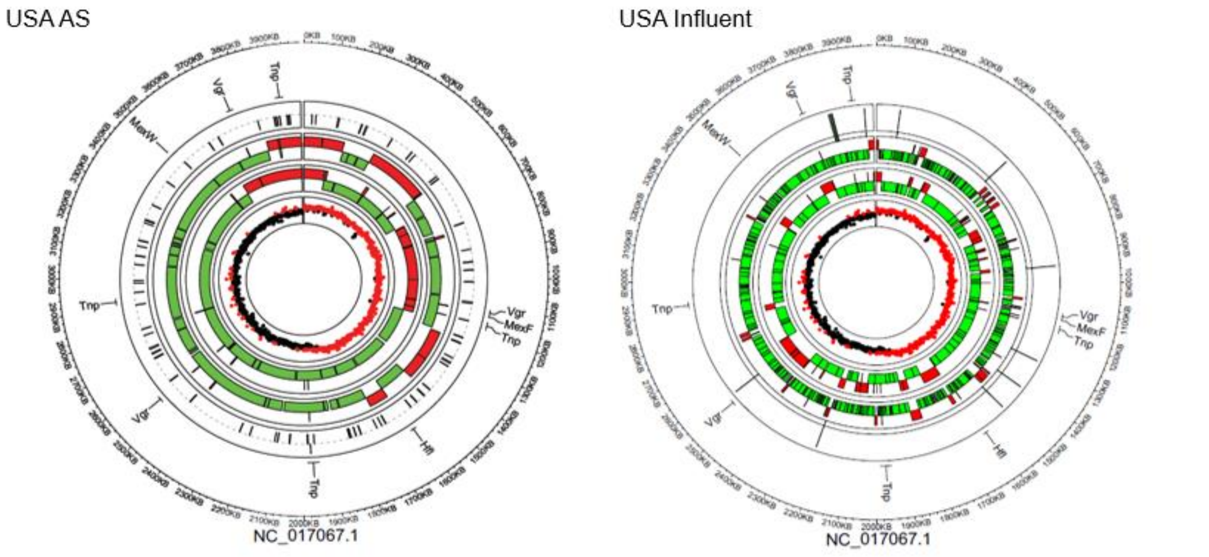

Figure S6. Hits to reference genome from Canu assemblies of the *in silico* spiked metagenomes. From outside towards the inside: 0.1x (misassembly), 0.1x (correct assembly); 1x, 3x (influent) or 5x (AS). Green indicates a contig that was correctly assembled. Red indicates a contig with a misassembly. Made with R(v3.5.0) and Circlize(v0.4.11).

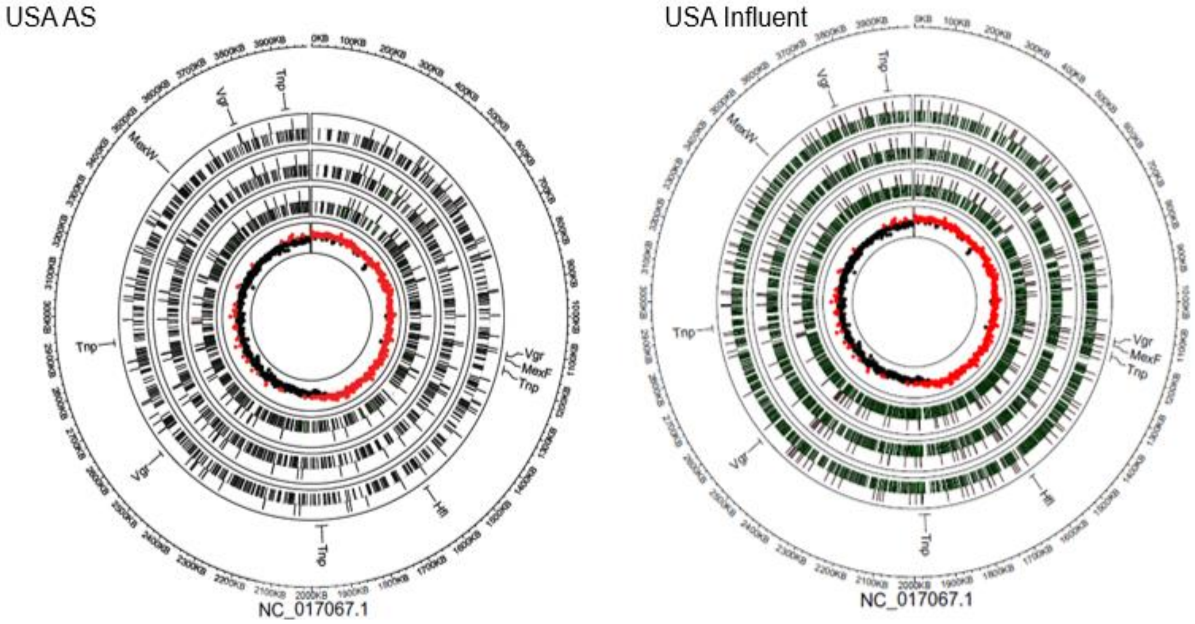

Figure S7. Hits to reference genome from HybridSpades assemblies of the *in silico* spiked metagenomes, all with 5x coverage of the reference genome for short reads. Long read coverage from outside towards the inside: 0.1x (misassembly), 0.1x (correct assembly); 1x, 3x (influent) or 5x (AS). Green indicates a contig that was correctly assembled. Red indicates a contig with a misassembly. Made with R(v3.5.0) and Circlize(v0.4.11).

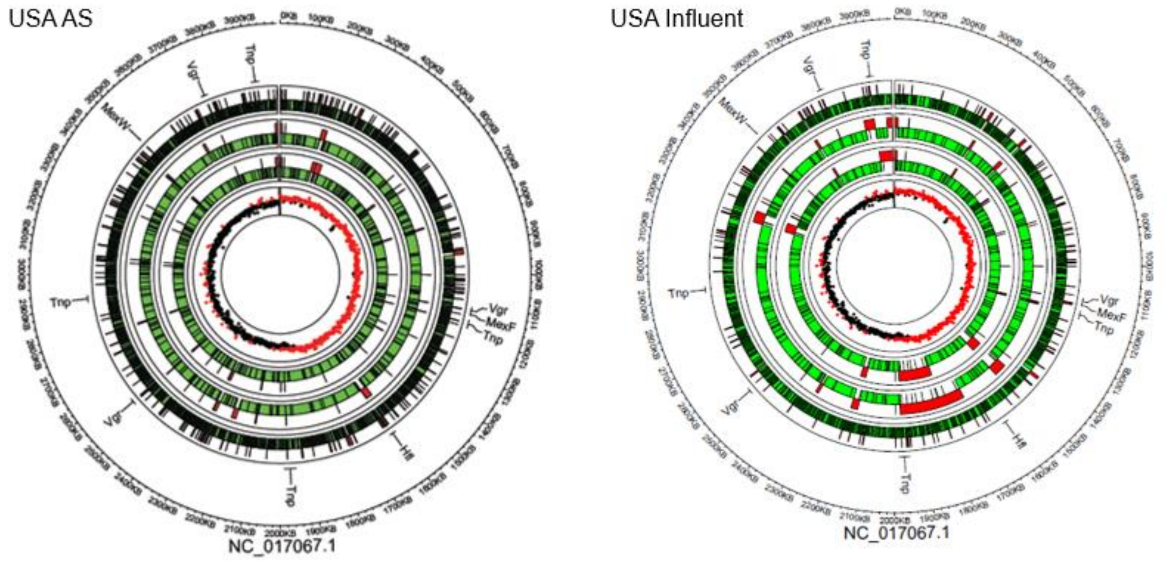

Figure S8. Hits to reference genome from HybridSpades assemblies of the *in silico* spiked metagenomes, all with 1x coverage of the reference genome for short reads. Long read coverage from outside towards the inside: 0.1x (misassembly), 0.1x (correct assembly); 1x, 3x (influent)

Made with R(v3.5.0) and Circlize(v0.4.11).or 5x (AS). Green indicates a contig that was correctly assembled. Red indicates a contig with a misassembly.

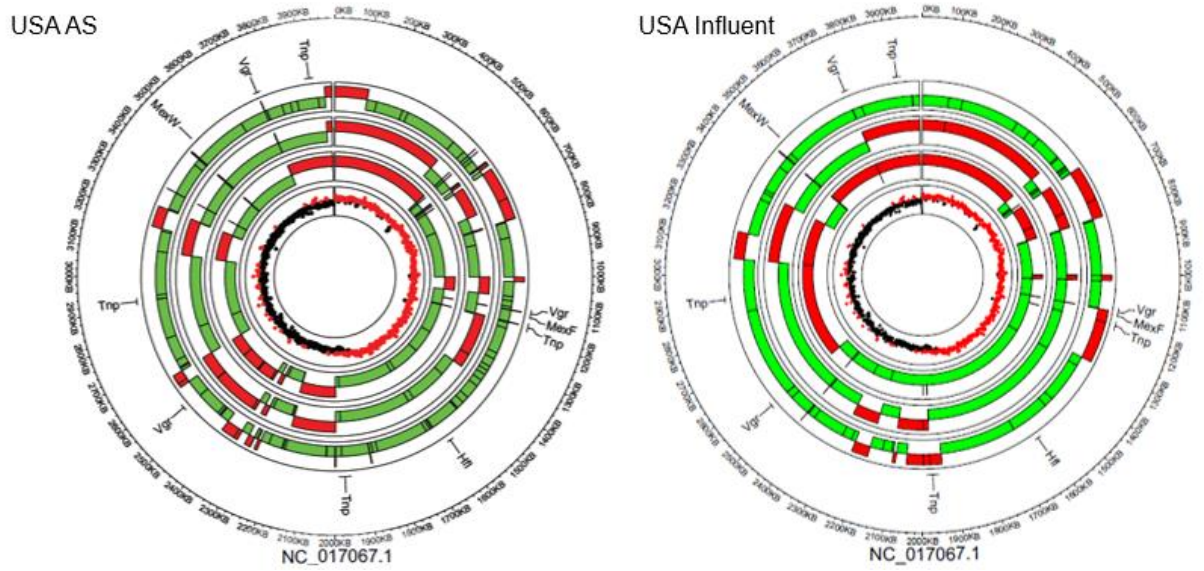

Figure S9. Hits to reference genome from HybridSpades assemblies of the *in silico* spiked metagenomes, all with 10x coverage of the reference genome for short reads. Long read coverage from outside towards the inside: 0.1x (misassembly), 0.1x (correct assembly); 1x, 3x (influent) or 5x (AS). Green indicates a contig that was correctly assembled. Red indicates a contig with a misassembly. Made with R(v3.5.0) and Circlize(v0.4.11).

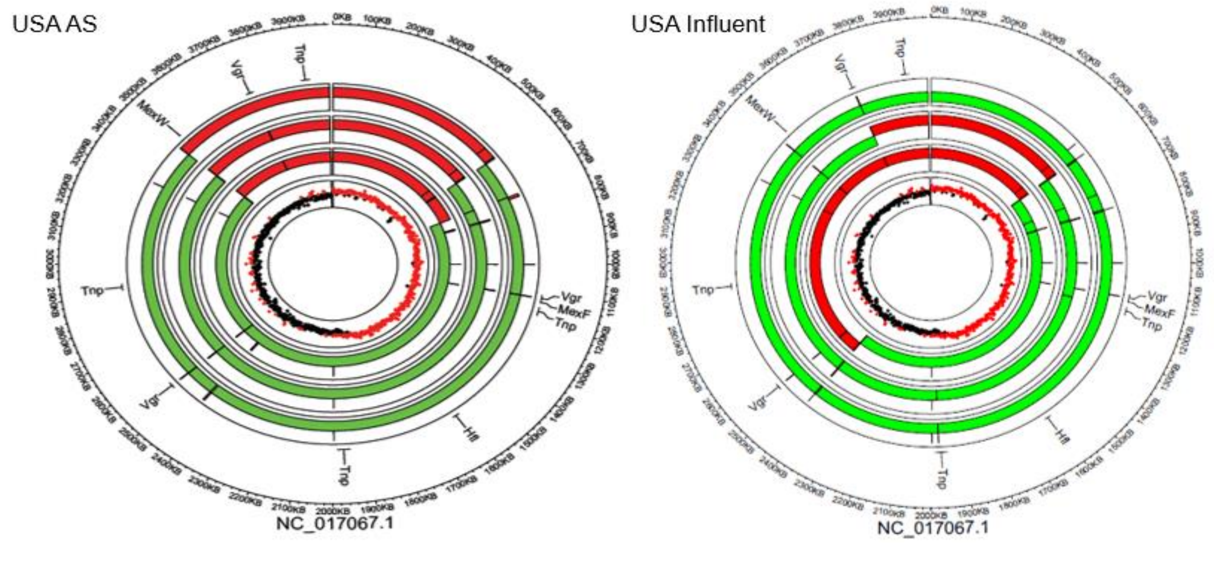

Figure S10. Hits to reference genome from HybridSpades assemblies of the *in silico* spiked metagenomes, all with 50x coverage of the reference genome for short reads. Long read coverage from outside towards the inside: 0.1x (misassembly), 0.1x (correct assembly); 1x, 3x (influent) or 5x (AS). Green indicates a contig that was correctly assembled. Red indicates a contig with a misassembly. Made with R(v3.5.0) and Circlize(v0.4.11).

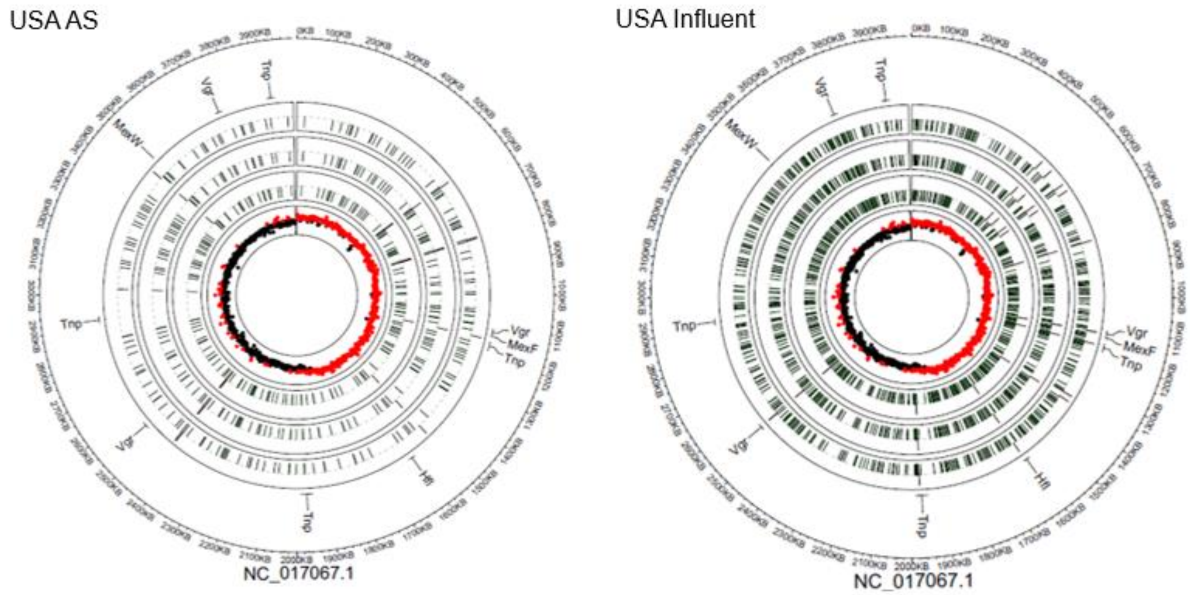

Figure S11. Hits to reference genome from Opera-MS assemblies of the *in silico* spiked metagenomes, all with 1x coverage of the reference genome for short reads. Long read coverage from outside towards the inside: 0.1x (misassembly), 0.1x (correct assembly); 1x, 3x (influent) or 5x (AS). Green indicates a contig that was correctly assembled. Red indicates a contig with a misassembly. Made with R(v3.5.0) and Circlize(v0.4.11).

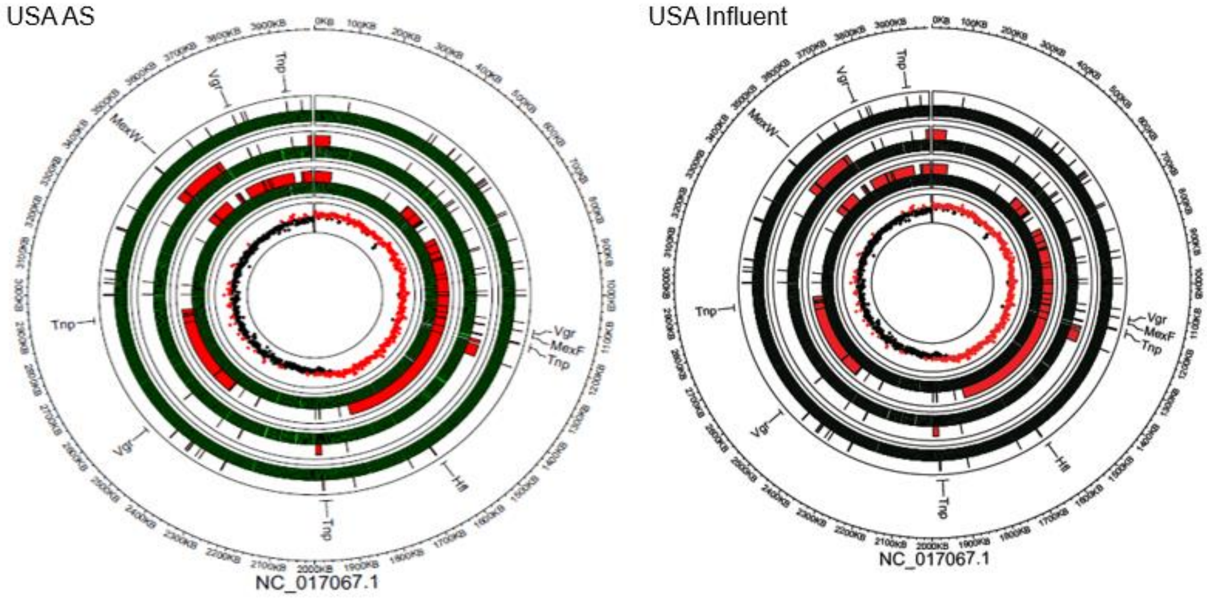

Figure S12. Hits to reference genome from Opera-MS assemblies of the *in silico* spiked metagenomes, all with 5x coverage of the reference genome for short reads. Long read coverage from outside towards the inside: 0.1x (misassembly), 0.1x (correct assembly); 1x, 3x (influent).

Made with R(v3.5.0) and Circlize(v0.4.11).or 5x (AS). Green indicates a contig that was correctly assembled. Red indicates a contig with a misassembly.

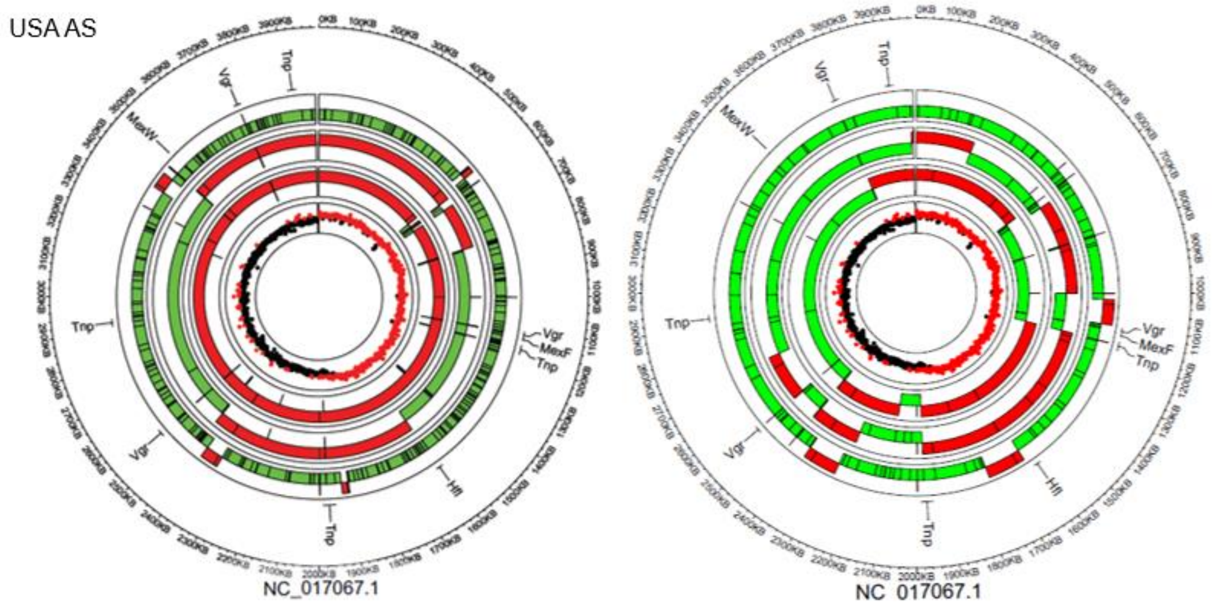

Figure S13. Hits to reference genome from Opera-MS assemblies of the *in silico* spiked metagenomes, all with 10x coverage of the reference genome for short reads. Long read coverage from outside towards the inside: 0.1x (misassembly), 0.1x (correct assembly); 1x, 3x (influent) or 5x (AS). Green indicates a contig that was correctly assembled. Red indicates a contig with a misassembly. Made with R(v3.5.0) and Circlize(v0.4.11).

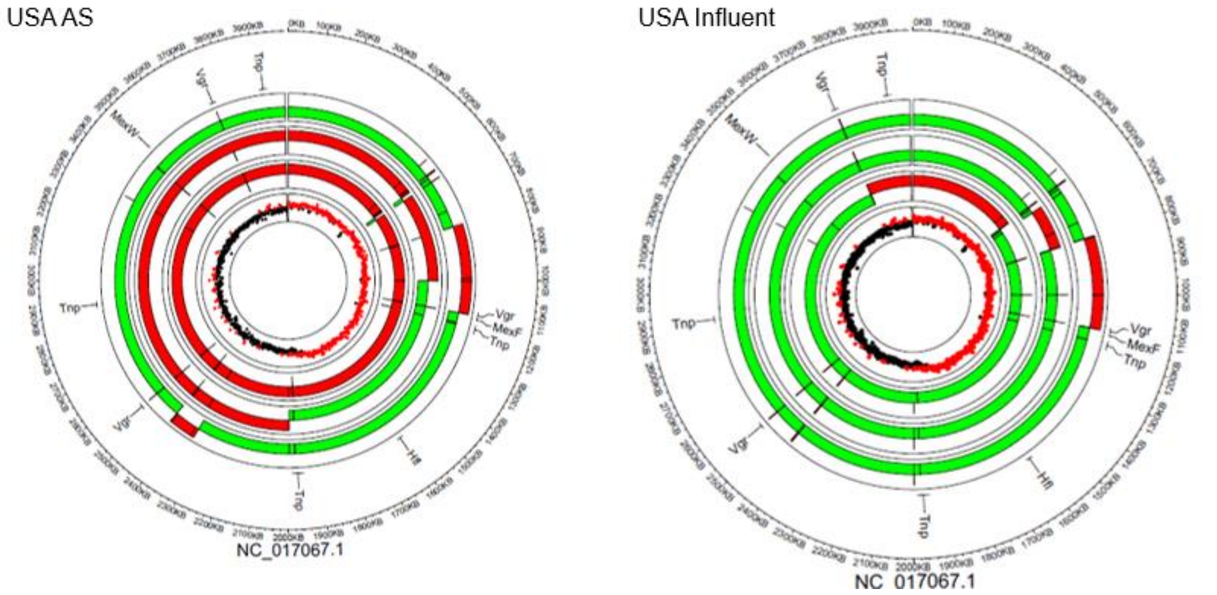

Figure S14. Hits to reference genome from Opera-MS assemblies of the *in silico* spiked metagenomes, all with 50x coverage of the reference genome for short reads. Long read coverage from outside towards the inside: 0.1x (misassembly), 0.1x (correct assembly); 1x, 3x

(influent) or 5x (AS). Green indicates a contig that was correctly assembled. Red indicates a contig with a misassembly. Made with R(v3.5.0) and Circlize(v0.4.11).

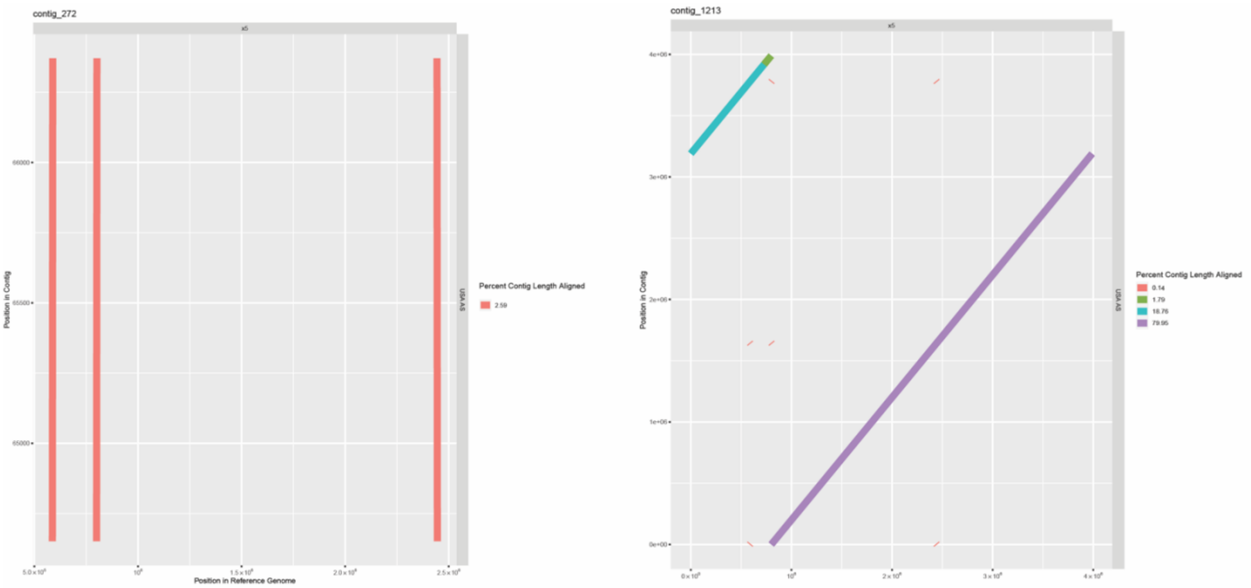

Figure S15: Dotplot of 5x metaFlye contigs of the USA-AS sample visualized in the circular genome plot in the main text (Fig. 5d). Made with R(v3.5.0) and ggplot2(3.3.0).
