# Supplementary material for: Critical evaluation of short, long, and hybrid assembly for contextual analysis of antibiotic resistance genes in complex environmental metagenomes
Source: Sci Rep. 2021 Feb 12;11:3753. doi: 10.1038/s41598-021-83081-8 (PMC7881036; doi:10.1038/s41598-021-83081-8)
Supplement: Supplementary file 2 — Supplementary Information 2. [file 41598_2021_83081_MOESM2_ESM.pdf]

Supplemental Information 2:

Critical evaluation of short, long, and hybrid assembly for contextual analysis of antibiotic resistance genes in complex environmental metagenomes

Authors: *Connor Brown[1]*, Ishi Keenum[2]*, Dongjuan Dai[2], Liqing Zhang[1], Peter Vikesland[2], Amy Pruden[2]*

[1]Virginia Tech, Genetics, Bioinformatics, and Computational Biology, Blacksburg, VA 24060
[2]Virginia Tech, Department of Civil & Environmental Engineering, Blacksburg, VA 24060

*These authors contributed equally to the work
Corresponding author: : Amy Pruden and Liqing Zhang

Software packages used:
IDBA-UD (September 2019)
metaSPAdes, hybridSPAdes (v 3.14.1)
MEGAHIT (v 1.2.8)
Canu (v1.8)
metaFlye(v2.6)
OPERA-MS(v0.8.3)
NanoSim (v 2.5.1)
Nucmer(v 3.3)
R(v 3.5)
Vegan (v 2.5-5)

Assembler specific commands:

1. IDBA-UD ./idba_ud -o ${sample} -r / ${sample}.merged.fa
2. Megahit ./megahit -1 /${sample}_1.fastq.gz -2 /${sample}_2.fastq.gz --presets meta-large -o /${sample} --out-prefix ${sample}
3. Canu ./canu \ -p ${sample} -d /${sample}corMinCoverage=0 corOutCoverage=all corMhapSensitivity=high correctedErrorRate=0.105 genomeSize=5m corMaxEvidenceCoverageLocal=10 corMaxEvidenceCoverageGlobal=10 oeaMemory=32 redMemory=32 batMemory=200 useGrid=false \ -nanopore-raw ./${sample}.fasta
4. MetaFlye ./flye_metaflye --nano-raw ${sample} --genome-size 1g --threads $(nproc) --meta --out-dir flye_meta_1g
5. HybridSPADES ./spades.py --meta --nanopore ${sample} --pe1-1 ${sample_illumina_r1} --pe1-2 ${sample_illumina_r2}-o ${sample}
6. OPERA-MS ./OPERA-MS.pl --short-read1 /illumina/${sample}_1.fastq.gz --short-read2 ./illumina/${sample}_2.fastq.gz \ --long-read ./nanopore.data/${sample}.fasta \ --out-dir /${sample}
7. metaSpades ./SPAdes-3.14.1-Linux/bin/spades.py --meta -1 ./illumina/${sample}_1.fastq.gz -2 ./illumina/${sample}_2.fastq.gz -t 96 -o ./Assembled_data/${sample}_metaSpades

Nucmer output filtering

Packages used:

```
library(ggplot2)
library(Rmisc)
library(ggpubr)
library(gridExtra)
library(data.table)
library(tidyr)
library(dplyr)
library(stringr)
library(forcats)
library(circlize)
```

Code description:

1. readCoords:Read in lists of Nucmer .coord data frames (note: some changing of the string manipulation in the function might need to happen)
2. sortContigs:Sort processed Nucmer coord file to detect misassemblies
3. summarisesorted: summarise data frames to calculate misassembly statistics

```
readCoords=function(x){
  nucmer=fread(as.character(x), header=FALSE)
  nucmer$SAMPLE=as.character(x)
  nucmer$SAMPLE=gsub('delta.coords','',nucmer$SAMPLE)
  nucmer$SAMPLE=gsub('nucmer.coords','',nucmer$SAMPLE)
  nucmer$SAMPLE=gsub('_','',nucmer$SAMPLE)
  nucmer$SAMPLE=gsub('HYBRIDSPADES','',nucmer$SAMPLE)
  nucmer$SAMPLE=as.factor(nucmer$SAMPLE)
  names<-c('S1','E1',
           'S2','E2',
           'LEN1','LEN2',
           '%ID','LENR',
           'LENQ','COVR',
           'COVQ','RG','CTG','SAMPLE')
  setnames(nucmer,names)
  nucmer$CTG=as.factor(nucmer$CTG)
  return(nucmer)
}

sortContigs=function(DF){
#inner functions:
#findoverlaps: looks for overlaps by matching less than greater than statements for contigs with multiple alignments
#find inversions: this is run on multiple alignment data frame (after overlap code) and looks for directionality in the alignments using a slope calculation (negative + positive slopes in the same alignment indicate inversions)
#find indels: simple function on the out put of findoverlaps->findinversions->looks for sequences whose euclidean distance between multiple alignments is unequal to the euclidean distance between alignments in reference genome
# findoverlaps=function(x){
#    # x=OLDF#test
#     ctg_list=unique(levels(x$CTG))
#    # y=ctg_list[10]#test
#
#     innerfunction=function(y){ # inner function that gets applied to all contigs...
#     df=x%>%subset(CTG==as.character(y))#make ctg data frame
#
#     df <- cbind(Row.Names = rownames(df), df) #add row names
#     overlaps=x[0,] # make empty overlap data frame
#
#     checklist=c("")
```

```
#
#     for (i in df$Row.Names){#FIRST FOR LOOP: cycle through ref. contigs
#     #i=1#test
#     df.i=subset(df,Row.Names=as.character(i))%>%droplevels
#     df.TEST=subset(df,Row.Names!=as.character(i))%>%droplevels
#     # teststatement=paste("REF_ROW:", i,sep=" ")
#     # print(teststatement)
#
#      for (j in df.TEST$Row.Names){ # NESTED FOR LOOP: cycle through all comparisons
#
#        df.TEST.j=subset(df.TEST,Row.Names==as.character(j))#make test data frame
#
#      #teststatement2=paste("TEST_ROWS:",j,sep="") # test statements to check if for loop is
working
#      #print(teststatement2)
#
#
ifelse((df.i$CTG_START<=df.TEST.j$CTG_START)&(df.i$CTG_END>=df.TEST.j$CTG_ST
ART),
#            (checklist=paste("true",checklist,sep=" ")),
#            (checklist=paste("false",checklist,sep=" ")))}
#
#     if(grepl("true",checklist)==TRUE){overlaps=rbind(overlaps,df)}
#     return(overlaps)}
#     }
#
#     out=ldply(lapply(ctg_list,innerfunction))
#     return(out)
#
#   }
# findinversions=function(x){
#     #x is is the data frame to check
#     x=x%>%droplevels
#     y=x[0,]
#     list=unique(levels(x$CTG))
#
#     if (nrow(x)>0){
#     for (j in list){
#
#       #j='NODE_14804_length_589_cov_4.179775'
#       x.1=subset(x,CTG==as.character(j))
#       pos=x.1%>%subset(SLOPE>0&min(LEN2>0.1*LENQ))%>%droplevels
#       #print(pos)
#       neg=x.1%>%subset(SLOPE<0&min(LEN2>0.1*LENQ))%>%droplevels
#       #print(neg)
#       if (nrow(pos)==0 | nrow(neg)==0
```

```
#
#          # rownames(x.1[which.min(x.1$S1),]) == rownames(x.1[which.min(x.1$S2),]) &
#          # rownames(x.1[which.max(x.1$S1),]) == rownames(x.1[which.max(x.1$S2),])
#
#          ) {
#        x<-x
#        } else{
#        x<-x[!(x$CTG==as.character(j)),]
#        y=rbind(x.1, y)}}}
#
#        x=x%>%droplevels
#        y=y%>%droplevels
#        #return(x)
#        return(y)
#   }
# findindels=function(j){
#     j.1=j#j.1=j%>%subset(LEN2>0.1*LENQ)%>%droplevels
#     j.1$CTG_DIST=sqrt(((max(j.1$S2)-min(j.1$S2))^2)+((max(j.1$E2)-min(j.1$E2)))^2)
#     j.1$RG_DIST=sqrt(((max(j.1$S1)-min(j.1$S1))^2)+((max(j.1$E1)-min(j.1$E1)))^2)
#     out=j.1%>%subset(RG_DIST>(CTG_DIST+1000))
#     return(out)
# }

  #y=hybridspades # TEST DATA
  #process data frame to add variables that will be used below
  sample_list=unique(levels(DF$SAMPLE))
  unique(levels(fmlrc2$SAMPLE))

 # DF=fmlrc2
INNERFX=function(A){
   # DF=shorts
   # A="metaSPAdes_USA INF_10x"
#A="FMLRC_USA AS_x10 x 5x"
  y=DF%>%subset(SAMPLE==as.character(A))
  y$SLOPE=(y$E2-y$S2)/(y$E1-y$S1) #write slope variable
  x=droplevels(subset(y, `%ID`>=95 & LEN2>=210))%>%droplevels #filter out background
alignments
  # x=x%>%rowwise()%>% mutate(CTG_START = min(S2,
E2),CTG_END=CTG_START+LEN2)
  # x=x%>%rowwise()%>% mutate(CTG_START = min(S1,
E1),REFG_END=REFG_START+LEN1)
  good=x%>%subset(COVQ>=99)%>%droplevels()#"good" contigs are those with query
coverage >= 99
  x=x%>%filter(CTG%notin%good$CTG)%>%droplevels()

    #Work with alignments
```

```
    multiple_aln_list=x%>%count(CTG,sort=TRUE)%>%subset(n>1)%>%droplevels() #get
those contigs with multiple alignments
    multiple_alns=x%>%filter(CTG%in%multiple_aln_list$CTG)%>%droplevels() #make new
df with those contigs

    presumptive.chimeras=x%>%
      filter(CTG%notin%multiple_aln_list$CTG)%>%
      droplevels()#all those left are singlets with COVQ<99: presumptive chimeras
      chimeras.mals=multiple_alns%>%group_by(CTG)%>%filter(sum(COVQ)<99)%>%
        droplevels()%>%ungroup
      presumptive.chimeras=rbind(presumptive.chimeras,chimeras.mals)%>%droplevels()

multiple_alns=multiple_alns%>%filter(CTG%notin%presumptive.chimeras$CTG)%>%droplev
els()


   # set aside those contigs that align over the end of the genome.
    endCtg_list=multiple_alns%>%subset(E1=="3989480")%>%droplevels
      endCtgs=multiple_alns%>%filter(CTG%in%endCtg_list$CTG)%>%droplevels

multiple_alns=multiple_alns%>%filter(CTG%notin%endCtg_list$CTG)%>%droplevels#clean
end-ctgs out of MAL df

    #
covq_too_low=multiple_alns%>%group_by(CTG)%>%filter(sum(COVQ)<99)%>%droplevels(
)%>%ungroup
    #
multiple_alns=multiple_alns%>%filter(CTG%notin%covq_too_low$CTG)%>%droplevels()
    #   presumptive.chimeras=rbind(covq_too_low,presumptive.chimeras)
    # #next check for overlapping alignments. at this stage, we have removed identical alignments,
or those that
    # #cannot be good alignments because the sum of the query coverages is less than 100.
    # overlapDF_check=multiple_alns
    #   overlapDF_check=overlapDF_check%>%subset(COVQ>1)%>%droplevels()
    #   overlaps=findoverlaps(overlapDF_check)%>%droplevels
    #   multiple_alns=multiple_alns%>%filter(CTG%notin%overlaps$CTG)%>%droplevels
    #next, count indels in the mutliple align data frame and in the overlap dataframe.
    # overlaps_INDELs=findindels(overlaps)
    # overlaps_INDELs$ASSEMBLY_TYPE="OVERLAP + INDEL"
    #
    # overlaps_INVERSIONS=findinversions(overlaps)%>%droplevels()
    # overlaps_INVERSIONS$ASSEMBLY_TYPE="OVERLAP + INVERSION"
    #
    # MA_INDELs=findindels(multiple_alns)%>%droplevels()
    # MA_INDELs$ASSEMBLY_TYPE="INDEL"
    #
```

```
   # MA_INVERSIONS=findinversions(multiple_alns)%>%droplevels()
   # MA_INVERSIONS$ASSEMBLY_TYPE="INVERSION"
    #endCtgs=presumptive.chimeras

if(nrow(presumptive.chimeras)>0){presumptive.chimeras$ASSEMBLY_TYPE="CHIMERA"}

    if(nrow(multiple_alns)>0){multiple_alns$ASSEMBLY_TYPE="MULTI-ALIGN"}

    if(nrow(endCtgs)>0){endCtgs$ASSEMBLY_TYPE="End Contig"}

    if(nrow(good)>0){good$ASSEMBLY_TYPE="Good"}

    # ifelse(nrow(endCtgs)>0, (endCtgs$ASSEMBLY_TYPE="End Contig"&
    #                         out=rbind(endCtgs,presumptive.chimeras,good,multiple_alns)),
    #                         (out=rbind(presumptive.chimeras,good,multiple_alns)))
    out=rbind(presumptive.chimeras,endCtgs,good,multiple_alns)
    return(out)}

  final_out=ldply(lapply(sample_list,INNERFX))
  return(final_out)
}

summariseSorted.1=function(x){
#x=sortContigs(flye)

sample_list=unique(levels(x$SAMPLE))

innerfx=function(y){
 # y=sample_list[1]
  ctdf=x%>%subset(SAMPLE==as.character(y))%>%droplevels()
  SAMPLE=unique(levels(ctdf$SAMPLE))
  ASSEMBLER=unique(levels(ctdf$ASSEMBLER))
  SITE=unique(levels(ctdf$SITE))
  COVERAGE=unique(levels(ctdf$COVERAGE))

  ctdf=unique(data.frame(ctdf$CTG,ctdf$LENQ,
                   ctdf$SAMPLE,ctdf$ASSEMBLER,
                   ctdf$SITE,ctdf$COVERAGE,
                   ctdf$SHORT,ctdf$LONG))%>%droplevels()

colnames(ctdf)=c("CTG","LENQ","SAMPLE","ASSEMBLER","SITE","COVERAGE","SHOR
T","LONG")


good_df=x%>%subset(SAMPLE==as.character(y)&ASSEMBLY_TYPE=="Good")%>%drople
vels()
```

```
bad=x%>%subset(SAMPLE==as.character(y)&ASSEMBLY_TYPE=="CHIMERA"|ASSEMB
LY_TYPE=="MULTIPLE ALIGN")%>%droplevels()

  BAD=length(unique(levels(bad$CTG)))
  GOOD=length(unique(levels(good_df$CTG)))

  BADBP=ctdf%>%filter(CTG%in%bad$CTG)
  GOODBP=ctdf%>%filter(CTG%in%good_df$CTG)

  BADBP=sum(BADBP$LENQ)
  GOODBP=sum(GOODBP$LENQ)

  TOTALBP=sum(BADBP+GOODBP)
  TOTAL=(BAD+GOOD)
  MPMBP=BAD/(TOTALBP/1e6)
  CTG_DIST=sort(ctdf$LENQ)
  CUMSUM=cumsum(ctdf$LENQ)
  n50df=data.frame(CTG_DIST,CUMSUM)
  N50=n50df[n50df$CUMSUM > max(n50df$CUMSUM)/2,][1,]
  N50=N50$CTG_DIST
  SHORT=ctdf$SHORT
  LONG=ctdf$LONG



out=data.frame(BAD,GOOD,TOTAL,SAMPLE,COVERAGE,ASSEMBLER,SITE,GOODBP,B
ADBP,TOTALBP,MPMBP,N50,SHORT,LONG)

  # colnames(out)=c("BAD","GOOD","TOTAL","SAMPLE",
  #                "COVERAGE","ASSEMBLER",
  #                "SITE","GOODBP","BADBP","TOTALBP",
  #                "MPMBP","N50")
  out$SAMPLE=as.factor(out$SAMPLE)
  out$COVERAGE=as.factor(out$COVERAGE)
  out$ASSEMBLER=as.factor(out$ASSEMBLER)
  out$SITE=as.factor(out$SITE)
  return(out)
}
final_out=ldply(lapply(sample_list,innerfx))
return(final_out)
}
```
